# Associations Between Early Screen Exposure and Neurocognitive Outcomes in Children Aged 2–5 Years: A Systematic Review

**DOI:** 10.3390/healthcare14183081

**Published:** 2026-09-19

**Authors:** Gabriela Georgieva Panayotova, Dilyara Nihatova Yakabova

**Affiliations:** 1 Division of Physiology, Department of Physiology and Pathophysiology, Medical University of Varna, 9002 Varna, Bulgaria; 2 Faculty of Medicine, Medical University of Varna, 9002 Varna, Bulgaria; dilyaranihatova05@gmail.com

**Keywords:** screen exposure, screen time, preschool children, early childhood development, language development, cognitive development, executive function, attention, caregiver co-viewing

## Abstract

**Highlights:**

**What are the main findings?**

- This systematic review of 43 quantitative studies found that greater screen exposure in children aged 2–5 years was most consistently associated with less favourable language, attention-regulation, and selected executive-function outcomes.
- Associations with general cognitive performance were more heterogeneous and varied according to media content, background exposure, caregiver co-viewing, family routines, and the home learning environment.

**What are the implications of the main findings?**

- Early childhood media guidance should address not only screen duration, but also content quality, passive and background exposure, caregiver involvement, and whether screen use displaces sleep, play, reading, and interaction.
- Family-centred, non-stigmatising recommendations and stronger longitudinal and intervention studies are needed to clarify which media-use contexts carry the greatest developmental relevance.

**Abstract:**

**Background/Objectives:** Screen exposure is widespread in preschool children, but developmental associations are difficult to interpret because use varies by duration, content, and caregiving context. This review synthesized evidence on associations with language development, general cognitive performance, attention regulation, and executive functioning in typically developing children aged 2–5 years. **Methods:** PubMed, Web of Science, and Scopus were searched for peer-reviewed English-language quantitative studies published between January 2000 and December 2025. Study selection followed PRISMA 2020. Risk of bias was assessed using design-specific JBI critical appraisal tools. Of 1105 records identified, 860 remained after duplicate removal; 115 full texts were assessed, and 43 studies were included. Findings were narratively synthesized because of heterogeneity in exposure definitions, outcome measures, study designs, and effect estimates. **Results:** Greater screen exposure was generally associated with less favourable language outcomes, particularly expressive language, vocabulary, communication, and developmental measures. Attention regulation, self-regulation, and selected executive-function outcomes also showed adverse associations; findings for inhibitory control, working memory, cognitive flexibility, and general cognitive performance were more heterogeneous. Passive viewing, background media, solitary use, and highly stimulating entertainment-oriented content were more often associated with less favourable outcomes. Caregiver co-viewing, joint media engagement, and developmentally appropriate content were associated with weaker adverse or neutral findings. Most studies relied on parent-reported exposure and observational designs. Given the predominance of observational evidence, these associations should not be interpreted as causal, and residual confounding and reverse causality remain possible. **Conclusions:** Screen exposure in children aged 2–5 years is associated with neurocognitive outcomes in a context-dependent manner. Duration alone is insufficient; content, background exposure, caregiver involvement, family routines, and the home learning environment should also be considered. Findings support cautious, family-centred guidance and stronger longitudinal and intervention studies with more precise exposure measurement.

## 1. Introduction

### 1.1. Early Childhood Neurocognitive Development

Early childhood, particularly the period between 2 and 5 years of age, is characterized by rapid and highly dynamic neurodevelopment. During these preschool years, children acquire foundational language abilities, develop early cognitive skills, and begin to establish core executive functions, including attention regulation, inhibitory control, working memory, cognitive flexibility, and goal-directed behaviour. These capacities are fundamental to later learning, school readiness, socio-emotional functioning, and mental health across the life course [1].

Language development during this period is particularly dependent on frequent and reciprocal social interaction. Rich linguistic input, joint attention, turn-taking, caregiver responsiveness, and opportunities for active verbal expression contribute to the development of expressive and receptive language skills [2]. Similarly, executive functions develop through play, structured routines, social interaction, and repeated opportunities to regulate attention and behaviour. Because these processes are still emerging during the preschool period, environmental experiences may be especially relevant to developmental trajectories [3].

### 1.2. Screen Exposure in Early Childhood

Digital media are now embedded in the everyday environments of many young children. Television, smartphones, tablets, computers, streaming platforms, and digital applications are frequently used in homes, childcare settings, and family routines. As a result, children may be exposed to screens not only through direct viewing or active device use, but also through background television, caregiver media use, and media consumption during meals, play, or bedtime routines [4,5].

The growing prevalence of screen exposure among preschool-aged children has raised concerns regarding its potential developmental implications [6]. Many children exceed recommended limits for recreational screen use, while the type, purpose, timing, and social context of screen exposure vary substantially between families [7]. However, screen exposure should not be regarded as a single, uniform behaviour. It may include passive television viewing, interactive educational applications, video calls, entertainment-oriented content, background media, or shared media use with caregivers. These distinctions are important because different forms of digital media may be associated with different developmental experiences and outcomes [8,9].

### 1.3. Potential Pathways Linking Screen Exposure and Neurocognitive Development

Several theoretical mechanisms have been proposed to explain how screen exposure may be associated with neurocognitive development in young children [8]. One commonly cited explanation is the displacement hypothesis, according to which time spent using screens may replace activities that are particularly important during early childhood, including caregiver–child interaction, shared reading, imaginative play, outdoor activity, sleep, and unstructured exploration. Reduced opportunities for conversation, play, and responsive interaction may be relevant to the development of language and executive functioning [10].

A second proposed pathway concerns the characteristics of screen content and the cognitive demands it places on young children. Fast-paced audiovisual content, frequent scene changes, highly stimulating entertainment media, and fragmented narratives have been hypothesized to affect attentional engagement, sustained concentration, and behavioural self-regulation in preschool-aged children. Background television may also be relevant, even when children are not actively watching, because it can interrupt play, reduce caregiver–child verbal exchanges, and fragment joint attention [4,8,11].

At the same time, potential associations may depend on the context in which media are used. Educational content, interactive applications, and caregiver co-viewing may provide opportunities for explanation, shared attention, language scaffolding, and active engagement [12]. In contrast, solitary viewing, passive consumption, background exposure, and high levels of screen use may be more likely to coincide with reduced social interaction or displacement of developmentally enriching activities [5]. These mechanisms remain theoretical and may not operate uniformly across children, families, media types, or developmental contexts.

### 1.4. Inconsistencies in the Existing Evidence

Although research examining screen exposure and child development has expanded substantially, the available evidence remains heterogeneous and difficult to interpret. Previous studies have reported associations between greater screen exposure and less favourable language development, attentional regulation, executive functioning, and general cognitive performance [12,13,14]. However, findings are not fully consistent, and the magnitude and direction of associations often vary according to study design, child age, exposure measurement, developmental outcome, and adjustment for potential confounding factors.

A major limitation of the literature is the lack of standardization in the measurement of screen exposure. Many studies rely on parent-reported estimates of daily screen time, which may be affected by recall bias, social desirability bias, and limited information about the specific media content or context of use [15]. Studies also vary in whether they distinguish television viewing from tablet or smartphone use, foreground from background exposure, passive from interactive use, or educational from entertainment content [5,8].

Neurocognitive outcomes are similarly assessed using diverse measures, including parent-reported questionnaires, developmental screening instruments, standardized language assessments, behavioural tasks, and composite cognitive scores. This variability limits direct comparison between studies and complicates quantitative pooling of findings [16]. In addition, associations between screen exposure and developmental outcomes may be influenced by socioeconomic status, parental education, home learning environment, caregiver mental health, parenting practices, sleep, childcare attendance, and baseline child developmental characteristics. Residual confounding and reverse causality therefore remain important considerations, particularly in cross-sectional studies [17].

Existing reviews have often included broad age ranges spanning infancy, preschool years, school-age children, and adolescents. Such approaches may obscure developmental patterns specific to the preschool period, when language, attention, and executive functions are undergoing rapid maturation [18,19]. Furthermore, studies frequently examine total screen time without adequately considering contextual characteristics such as exposure dose, developmental timing, device type, background media, content quality, passive versus interactive use, and caregiver co-viewing [9,20,21].

Several previous systematic reviews and meta-analyses have already demonstrated that developmental associations with screen use extend beyond total duration. Madigan et al. reported that greater screen quantity and background television exposure were associated with poorer language skills, whereas educational content and caregiver co-viewing were associated with more favourable language outcomes [22]. Bustamante et al. specifically examined executive functioning in children younger than 6 years and found no overall association between total screen time and executive functioning, emphasizing the importance of contextual and developmental moderators [23]. More recently, Mallawaarachchi et al. synthesized 100 studies involving children from birth to 5.99 years and quantitatively demonstrated that program viewing, background television, content characteristics, and co-use were differentially associated with cognitive and psychosocial outcomes [24]. These reviews establish that screen use is multidimensional rather than a uniform exposure.

### 1.5. Aim and Scope of the Present Review

Against this background, the present review was designed as a developmentally focused and updated synthesis rather than as the first review to examine contextual characteristics of screen use. Its distinctive scope is the restriction to typically developing children aged 2–5 years, corresponding specifically to the preschool developmental period, together with the integrated examination of language development, general cognitive performance, attention regulation, and executive functioning within the same review framework. In contrast to broader early-childhood reviews extending from infancy through the sixth year of life, the present review focuses on the preschool period and incorporates evidence published through December 2025. It also examines the consistency of findings across neurocognitive domains alongside exposure measurement, study design, risk of bias, residual confounding, and contextual characteristics of screen use.

Screen exposure includes total daily screen time, television viewing, smartphone and tablet use, computer use, digital media exposure, background television or background media, passive and interactive media use, content characteristics, and caregiver co-viewing [5,9,21]. Eligible studies may compare children with lower versus higher screen exposure, different screen-use thresholds, or different media-use contexts. The secondary objectives are to examine whether reported associations differ according to screen exposure dose, timing, type of device, passive versus interactive media use, background media exposure, content type, and caregiver co-viewing. The review also aims to identify methodological limitations, sources of heterogeneity, and gaps in the available evidence in order to inform future longitudinal, interventional, and policy-oriented research.

## 2. Materials and Methods

### 2.1. Review Design and Reporting Framework

**Figure 1:**
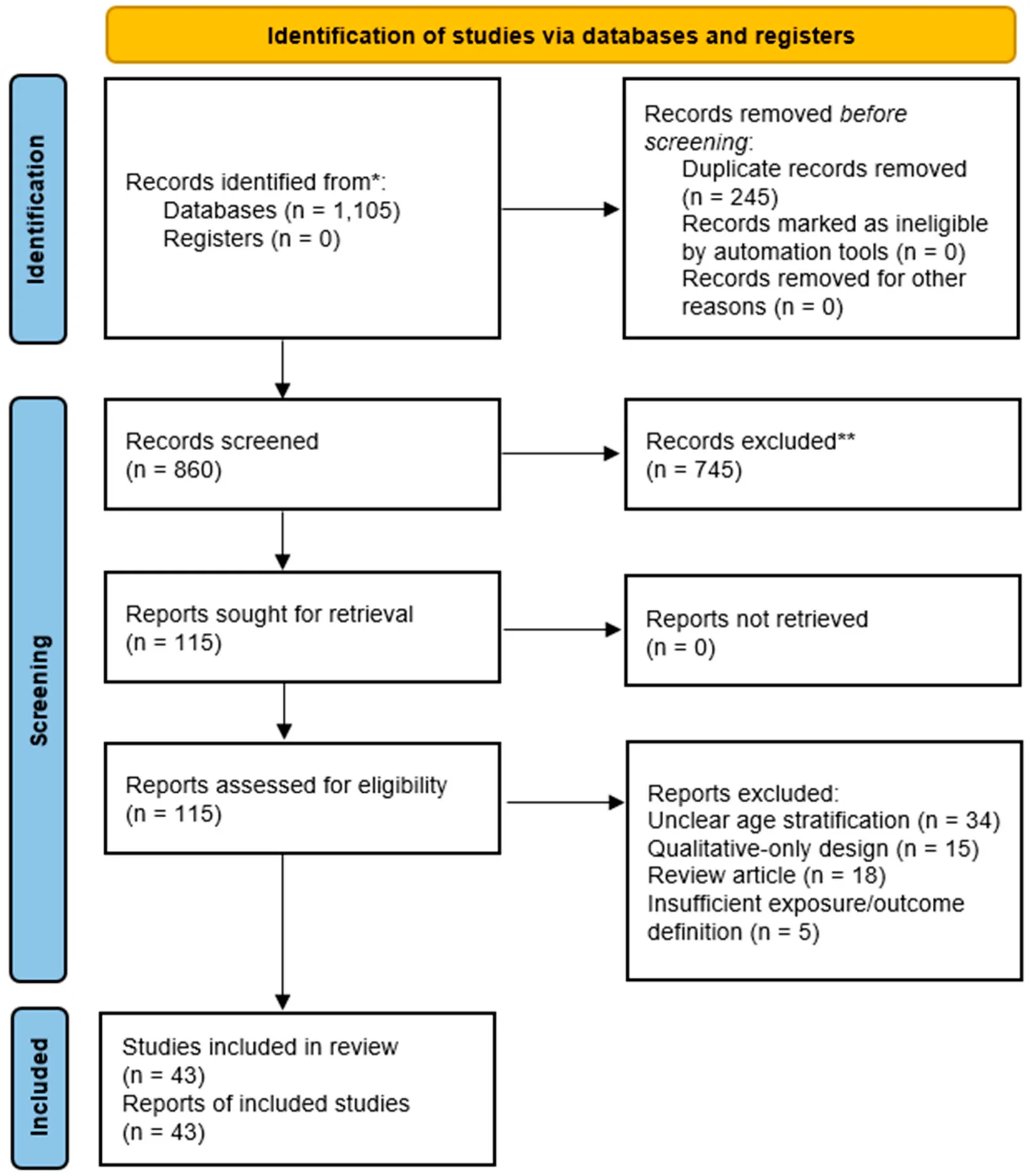
PRISMA flowchart showing studies identification, screening, and inclusion process. * Records were identified through database searching in PubMed, Scopus, and Web of Science; no registers were searched. ** Records were excluded during title and abstract screening because they did not meet the predefined eligibility criteria.

This systematic review examined associations between screen exposure and neurocognitive outcomes in typically developing children aged 2–5 years. The review was conducted and reported in accordance with the Preferred Reporting Items for Systematic Reviews and Meta-Analyses (PRISMA) 2020 statement. The study selection process is presented in a PRISMA 2020 flow diagram (Figure 1). This review was registered in the International Prospective Register of Systematic Reviews (PROSPERO; registration number CRD420261426450) on 17 June 2026. The review commenced on 9 January 2026, and formal searching and screening had been completed at the time of registration; therefore, the registration should not be interpreted as prospective registration. A separate full protocol was not published. The PROSPERO record is publicly available under registration number CRD420261426450. Any deviations from the registered methods are reported in Supplementary Table S1.

### 2.2. Eligibility Criteria

Eligibility criteria were defined before study selection and structured according to the population, exposure, comparator, outcome, and study-design framework.

#### 2.2.1. Population

Studies were eligible if they included typically developing children aged 2–5 years. Studies with wider age ranges were included only when data for children within the target age range were reported separately or could be independently extracted.

Studies focused exclusively on infants, school-aged children, adolescents, or adults were excluded. Studies involving children with diagnosed neurodevelopmental, neurological, psychiatric, genetic, or severe sensory disorders were also excluded unless data for typically developing children were separately reported.

#### 2.2.2. Exposure

Eligible studies quantitatively assessed screen exposure. Exposure measures included total daily screen time, television viewing, smartphone or tablet use, computer use, digital media exposure, background television or background media, passive screen viewing, interactive media use, and caregiver co-viewing.

Screen exposure could be reported as duration, frequency, categorical exposure level, or predefined daily threshold. Studies using parent-reported questionnaires, structured interviews, media-use diaries, or other quantitative exposure measures were eligible.

Studies were excluded when screen exposure was not clearly defined or quantified. Studies examining sedentary behaviour without a separate screen-exposure measure were also excluded.

#### 2.2.3. Comparators

Eligible studies compared children with different levels, durations, or contexts of screen exposure. Comparisons included lower versus higher exposure, minimal versus prolonged exposure, exposure below versus above recommended thresholds, and different media-use contexts.

Studies comparing passive versus interactive media use, foreground versus background media exposure, educational versus entertainment content, or caregiver co-viewing versus solitary viewing were also eligible.

#### 2.2.4. Outcomes

The review included studies assessing at least one neurocognitive outcome. Eligible outcomes included:

- expressive and receptive language development;
- vocabulary, language delay, and communication skills;
- general cognitive performance and developmental test scores;
- memory and problem-solving;
- attention regulation and sustained attention;
- inhibitory control;
- working memory;
- cognitive flexibility;
- self-regulation;
- broader executive functioning.

Outcomes could be measured using standardized developmental assessments, validated parent-reported questionnaires, developmental screening tools, clinical assessments, or performance-based behavioural tasks.

#### 2.2.5. Study Designs and Publication Characteristics

Eligible study designs included cross-sectional studies, longitudinal cohort studies, case–control studies, quasi-experimental studies, and interventional studies that quantitatively examined the association between screen exposure and at least one eligible neurocognitive outcome.

Only peer-reviewed studies published in English between 1 January 2000 and 31 December 2025 were included. Reviews, systematic reviews, meta-analyses, editorials, commentaries, letters, conference abstracts, protocols, grey literature, dissertations, qualitative-only studies, case reports, and case series were excluded.

### 2.3. Information Sources and Search Strategy

A systematic literature search was conducted in PubMed, Web of Science, and Scopus. The search strategy combined keywords related to screen exposure, early childhood, and neurocognitive development. Search terms included combinations of:

- “screen time”, “screen exposure”, “digital media”, “television”, “TV”, “smartphone”, “tablet”, “computer”, “background television”, and “background media”;
- “early childhood”, “preschool child”, “preschooler”, “toddler”, “young child”, and “children aged 2–5 years”;
- “language development”, “vocabulary”, “cognitive development”, “cognition”, “executive function”, “attention”, “inhibitory control”, “working memory”, “self-regulation”, and “developmental outcome”.

Boolean operators “AND” and “OR” were used to combine search concepts. Search strategies were adapted to the syntax and indexing structure of each database. All records were imported into Zotero version 7.0.32 (Corporation for Digital Scholarship, Vienna, VA, USA). Duplicate records were identified and removed before screening. No automation tools were used for record screening, eligibility determination, or study selection. The final database searches were conducted on 24 June 2026. The complete database-specific search strategies, including the exact syntax applied in PubMed, Web of Science, and Scopus, are provided in Supplementary Table S2.

### 2.4. Study Selection

Study selection was performed independently by two reviewers in two stages. First, titles and abstracts were screened against the predefined eligibility criteria (Figure 1). Second, the full texts of potentially eligible reports were independently assessed for final inclusion. Disagreements regarding study eligibility were resolved through discussion and consensus between the two reviewers.

The initial database search identified 1105 records. After 245 duplicate records were removed, 860 records remained for title and abstract screening. Of these, 745 records were excluded because they did not meet the eligibility criteria. A total of 115 full-text reports were sought and successfully retrieved for eligibility assessment.

Following full-text assessment, 72 reports were excluded because of unclear age stratification (*n* = 34), qualitative-only study design (*n* = 15), review article publication type (*n* = 18), or insufficient definition of screen exposure and/or neurocognitive outcomes (*n* = 5). A total of 43 studies were included in the final systematic review.

### 2.5. Data Extraction

Data were independently extracted from all included studies by two reviewers using a standardized extraction framework. Discrepancies were resolved through discussion and consensus. The following information was collected:

- first author and year of publication;
- country and study setting;
- study design;
- sample size;
- participant age range, mean age, and sex distribution;
- type and measurement method of screen exposure;
- screen-exposure duration, frequency, threshold, or category;
- outcome domain and measurement instrument;
- main findings and reported effect estimates;
- covariates included in adjusted analyses;
- study limitations reported by the authors;
- key methodological characteristics relevant to interpretation of the findings.

Where studies reported several outcomes or exposure categories, all relevant data were extracted. When multiple adjusted models were reported, emphasis was placed on estimates adjusted for major demographic and family-level confounding variables, including child age, sex, socioeconomic status, parental education, and home learning environment, where available. Study-level quantitative findings, including the principal reported effect estimates and corresponding measures of statistical uncertainty where available, are presented in Supplementary Table S3.

### 2.6. Methodological Quality and Risk-of-Bias Assessment

The methodological quality and risk of bias of the included studies were assessed using design-specific Joanna Briggs Institute (JBI) critical appraisal tools [25,26,27]. The revised JBI tools for cohort studies, analytical cross-sectional studies, and randomized controlled trials were applied according to the analytical design contributing to the present synthesis. For reports containing more than one analytical component, the appraisal tool was selected according to the component contributing the relevant neurocognitive association to this review. Each appraisal item was rated as Yes, No, Unclear, or Not applicable, as appropriate. No composite numerical quality score was calculated; instead, item-level judgments were used to identify methodological limitations relevant to evidence synthesis and interpretation. The appraisal was conducted independently by two reviewers, with disagreements resolved through discussion and consensus. Detailed study-level judgments and methodological concerns are presented in Supplementary File S2.

### 2.7. Data Synthesis

A narrative synthesis was conducted because of substantial heterogeneity across the included studies. Studies differed in screen-exposure definitions, exposure duration thresholds, device type, media context, outcome measures, participant characteristics, study design, and statistical approaches. Findings were grouped according to the following neurocognitive domains:

- language development;
- general cognitive performance;
- attention regulation;
- inhibitory control and working memory;
- broader executive functioning.

Within each domain, the evidence was further examined according to screen-exposure duration, type of device, passive versus interactive media use, background media exposure, content characteristics, and caregiver co-viewing, where these variables were reported. The direction, consistency, and strength of reported associations were considered alongside study design, covariate adjustment, and methodological limitations. Because outcome measures and exposure definitions were insufficiently comparable across studies, no meta-analysis was performed. The study-level quantitative estimates underlying the narrative synthesis, including the principal reported effect estimates and corresponding measures of statistical uncertainty where available, are provided in Supplementary Table S3.

### 2.8. Assessment of Evidence Strength and Certainty

The certainty of the evidence was evaluated using a structured, GRADE-informed narrative approach adapted to the heterogeneity of the included studies. For each major neurocognitive outcome domain, interpretation considered the JBI risk-of-bias findings, consistency of results across studies, directness of the evidence, precision of the reported associations, study design, temporal sequence, and the potential influence of residual confounding. Greater confidence was placed in convergent findings from prospective studies using standardized neurocognitive assessments, appropriate adjustment for major confounders, and fewer methodological concerns. Evidence derived predominantly from cross-sectional designs, caregiver-reported exposure measures, heterogeneous outcome assessments, small samples, or incompletely reported methodological procedures was interpreted with greater caution. Because substantial methodological and clinical heterogeneity precluded quantitative pooling, certainty was assessed narratively rather than through pooled estimates. GRADE-relevant domains were therefore used to qualify the interpretation of the evidence within each neurocognitive outcome domain.

## 3. Results

### 3.1. Characteristics of Included Studies

The 43 included studies were published between 2005 and 2025 and represented a geographically diverse evidence base, with studies conducted predominantly in North America, Europe, Australia, and East Asia. Study designs included cross-sectional investigations, longitudinal cohort studies, and randomized experimental studies examining short-term media effects. The country and study setting of each included study are reported in Supplementary Table S3; specific ethnic or population characteristics are reported where they were explicitly described in the original publications.

Sample sizes ranged from fewer than 100 participants in experimental and small cohort studies to several thousand children in large population-based cohorts. Most samples involved typically developing children during the preschool period, although several longitudinal studies assessed screen exposure during infancy or toddlerhood and examined neurocognitive outcomes between 2 and 5 years of age.

Screen exposure was assessed primarily through parent-reported questionnaires, structured interviews, media-use diaries, or caregiver estimates of average daily exposure. Any screen-exposure measure was assessed in all included studies, whereas television viewing was examined in 31 studies. Mobile-phone or tablet use was reported in 18 studies, background television or background media exposure in 12 studies, content type in 14 studies, and caregiver co-viewing in 9 studies. Several studies evaluated more than one screen-use dimension.

Language development was assessed in 30 studies, general cognitive performance in 18 studies, and executive functioning in 16 studies. Fourteen studies assessed more than one neurocognitive domain. Outcome measures included standardized language and developmental assessments, parent-reported developmental screening tools, behavioural tasks, executive-function measures, and questionnaires addressing attention, self-regulation, or behavioural functioning. The detailed characteristics of included studies are presented in Table 1, while the main screen-exposure dimensions and neurocognitive outcome domains are summarized in Table 2. The principal study-level quantitative findings and reported effect estimates are provided in Supplementary Table S3.

### 3.2. Screen Exposure and Language Development

Language development was the most frequently assessed outcome domain. Across the included studies, higher total screen exposure was generally associated with less favourable language outcomes, particularly expressive language, vocabulary, communication skills, and language-related developmental milestones [30,31,34,39,40,45,48].

Cross-sectional studies commonly reported that children with longer daily screen exposure had lower expressive vocabulary scores, poorer language performance, or a greater likelihood of language delay compared with children exposed to lower levels of screen use [30,34,39,40,48]. Similar patterns were reported in longitudinal studies, in which greater exposure during infancy, toddlerhood, or early preschool years was associated with less favourable language or communication outcomes at later assessments [30,31,35,45].

When expressive and receptive language were assessed separately, associations were generally more consistent for expressive language. Several studies reported that greater screen exposure was associated with poorer expressive vocabulary, verbal production, or communication skills, whereas findings for receptive language were more heterogeneous [34,39,40,48]. Some studies found weaker or non-significant associations after adjustment for parental education, socioeconomic factors, home literacy environment, or caregiver-child interaction [35,39,48].

The direction of association also differed according to media context. Passive television viewing, background television exposure, and solitary screen use were more often associated with less favourable language outcomes [34,35,40]. In contrast, shared media use with caregivers, educational content, and joint media engagement were associated with weaker adverse associations or, in some studies, neutral outcomes [28,34,39,40]. However, these potentially protective contextual characteristics were not assessed consistently across studies.

### 3.3. Screen Exposure and General Cognitive Performance

General cognitive performance was assessed in 18 studies through developmental screening instruments, composite developmental scores, problem-solving tasks, memory measures, and early educational skills. Overall, associations between screen exposure and general cognitive outcomes were less consistent than those observed for language development [29,32,33,36,38,45,47,51].

Several longitudinal studies reported that greater screen exposure was associated with lower developmental screening scores, poorer communication and problem-solving outcomes, or lower cognitive performance at follow-up [31,33,38,45,47,49,51]. For example, higher screen exposure at 24 and 36 months was associated with lower scores on developmental screening measures at subsequent assessments in a large longitudinal cohort [31]. Other studies similarly reported associations between earlier screen exposure and later communication delay, reduced problem-solving performance, or lower achievement of developmental milestones [33,45,47,49].

However, the magnitude and consistency of these associations varied substantially between studies. Associations were often attenuated after adjustment for socioeconomic status, parental education, home learning environment, caregiver interaction, baseline developmental characteristics, and other family-level variables [29,38,51,65]. Studies assessing interactive or educational media use reported mixed findings, with some indicating neutral or modestly favourable associations with selected cognitive outcomes, particularly when media use occurred in a developmentally supportive context.

Background media exposure was associated in several studies with reduced attention during play, poorer task engagement, or lower-quality caregiver-child interaction [41,66]. However, direct associations between background exposure and standardized global cognitive scores were examined less frequently than associations involving total screen time or television viewing.

### 3.4. Screen Exposure, Attention, and Executive Functioning

Executive functioning was examined in 16 studies, with attention regulation, inhibitory control, working memory, cognitive flexibility, and self-regulation representing the most frequently assessed outcomes. The findings indicated that greater screen exposure was commonly associated with less favourable attention and executive-function outcomes, although the strength of associations differed by study design, exposure type, and outcome measurement [53,54,57,58,62,63,67,68,69,70].

Observational studies generally reported that higher daily screen exposure was associated with more inattention symptoms, poorer self-regulation, reduced task persistence, and weaker performance on executive-function measures [57,58,62,63,67,69,70]. Associations were reported for both television viewing and broader screen-use measures, including mobile-device use and electronic gameplay [54,60,61,64,67,68]. In several studies, children exposed to greater amounts of screen time demonstrated higher levels of inattention or behavioural dysregulation after adjustment for major demographic and family-level variables [58,62,63,67,69].

Attention regulation was the most consistently examined executive-function outcome. Studies assessing screen time in preschool-aged children frequently reported associations between greater exposure and attentional difficulties, distractibility, or reduced sustained attention [58,62,63,64,69]. Threshold-based analyses suggested that unfavourable attention-related outcomes were more frequently reported among children exposed to more than 2 h of screen time per day, although exposure thresholds and outcome definitions varied across studies [58].

Evidence regarding inhibitory control, working memory, and cognitive flexibility was more heterogeneous. Some studies reported poorer task performance among children with higher levels of screen exposure, whereas others reported weak or non-significant associations after adjustment for contextual and familial variables [56,61,62,68]. Studies relying on direct behavioural tasks often produced more differentiated findings than studies using parent-reported measures of executive functioning [52,56,59].

Experimental studies examining short-term effects of screen content reported that executive-function performance differed according to the type of media exposure [52,55,56,59]. Exposure to specific fast-paced or fantastical content was associated with poorer immediate performance on selected executive-function tasks in some studies, whereas educational programming and interactive applications showed more variable effects [52,56,59]. These studies evaluated immediate task performance and should therefore be interpreted separately from longitudinal developmental associations.

### 3.5. Dose, Timing, and Persistence of Exposure

Across outcome domains, studies assessing screen exposure as a continuous or categorical variable generally reported less favourable outcomes at higher exposure levels [30,31,39,48,58,62]. The most consistent associations were observed in studies examining prolonged daily screen exposure, particularly when children exceeded approximately 2 h per day [30,58]. Associations at lower levels of exposure were more variable and were often influenced by the type and context of media use [28,35,39,40,65].

Several longitudinal studies indicated that earlier exposure, particularly during infancy and toddlerhood, was associated with later developmental outcomes during the preschool period [31,33,43,44,45,62]. Persistent or increasing screen-use trajectories across early childhood were also associated with less favourable language, cognitive, or executive-function outcomes in some cohort studies [51,70]. However, substantial variation in the timing of exposure measurement, duration of follow-up, and outcome assessment prevented direct comparison of specific developmental thresholds across the evidence base [31,38,45,51,70].

### 3.6. Contextual Characteristics of Screen Use

Contextual characteristics of screen exposure were assessed less frequently than total screen duration but contributed substantially to variability in findings. Studies examining content type generally reported less favourable outcomes in relation to passive, entertainment-oriented, or fast-paced media compared with educational, interactive, or age-appropriate content [28,29,37,52,55,56,59,65]. However, definitions of “educational” content differed between studies, and several investigations did not account for the quality or developmental appropriateness of the media assessed [29,37,39,65].

Caregiver co-viewing and joint media engagement were associated with more favourable language-related outcomes in studies that assessed these variables [28,35,39,40]. Co-viewing accompanied by explanation, questioning, and verbal interaction was generally associated with weaker adverse associations than solitary viewing [28,39,40]. In contrast, background television or background media exposure was commonly associated with reduced caregiver-child interaction, more fragmented play, and poorer attention-related outcomes [41,66].

**Figure 2:**
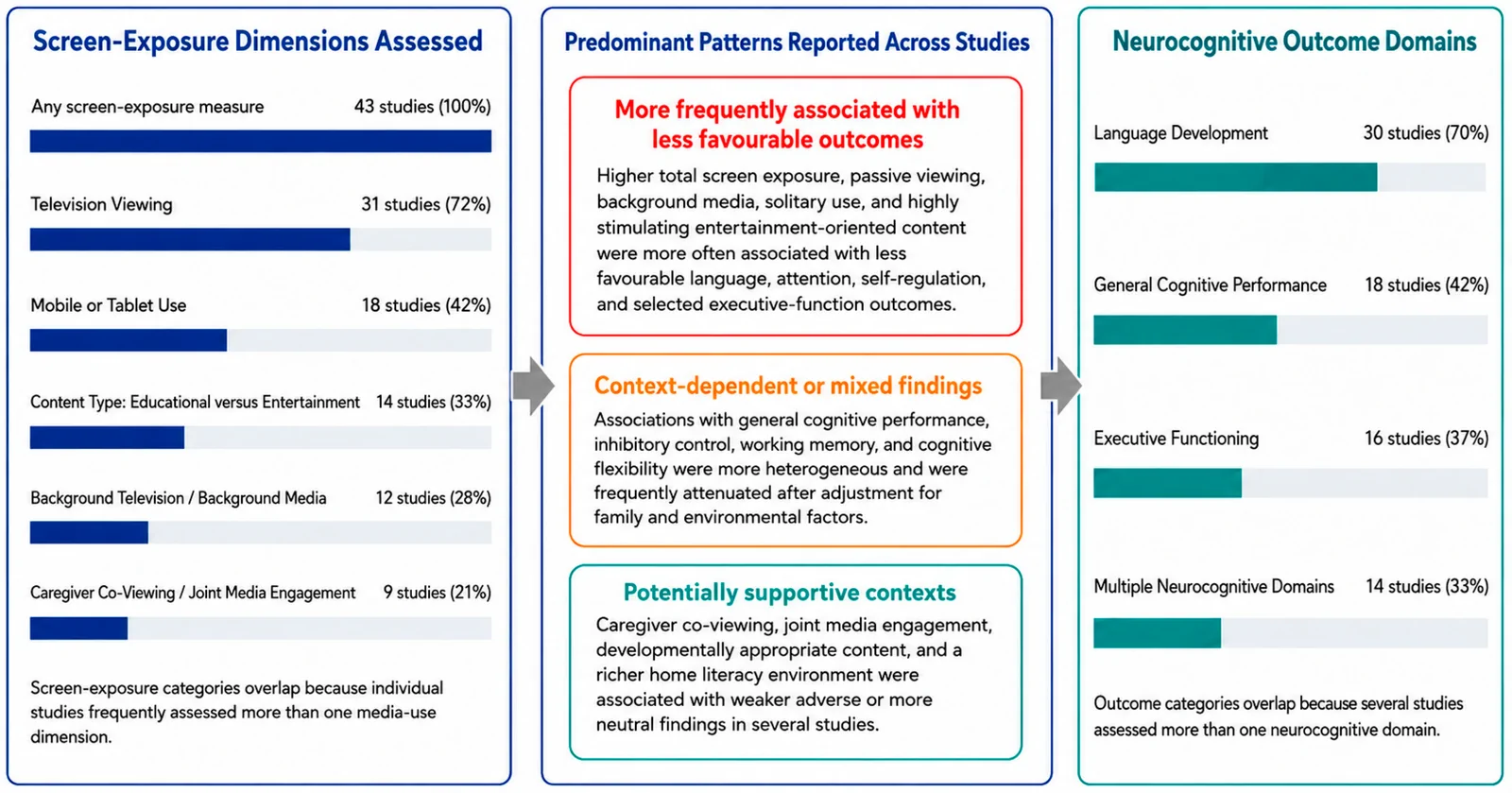
Evidence profile of screen-exposure dimensions, contextual characteristics, and neurocognitive outcomes across 43 included studies. Exposure and outcome categories were not mutually exclusive. The figure summarizes the predominant pattern of reported associations and does not establish causality.

The home learning environment and family socioeconomic characteristics were considered in many, but not all, studies [29,31,38,39,40,51,69]. Associations between screen exposure and neurocognitive outcomes were frequently reduced after adjustment for parental education, socioeconomic status, home literacy activities, caregiver mental health, parenting practices, and child-care routines (Figure 2) [31,35,39,40,48,69]. Nevertheless, higher screen exposure remained associated with less favourable outcomes in several adjusted analyses (Table 2) [30,31,45,48,51,58].

### 3.7. Methodological Characteristics of the Evidence Base

The included studies were characterized by substantial methodological heterogeneity. Screen exposure was most commonly measured through caregiver-reported estimates, although the operationalization of exposure varied widely [31,39,40,48,58,69]. Some studies examined total daily screen time, whereas others focused on television viewing, mobile-device use, background media exposure, media content, or caregiver co-viewing [28,29,35,39,56,64,66,67,68]. Exposure thresholds also differed, limiting direct comparison between studies [30,39,48,58,70].

Neurocognitive outcomes were assessed using a broad range of instruments, including standardized language tests, developmental screening measures, behavioural tasks, parent-reported questionnaires, and composite cognitive scores [30,31,32,42,45,46,47,50]. This variability was particularly pronounced for executive functioning, where studies differed in whether they assessed attention, working memory, inhibition, self-regulation, or broader behavioural functioning [53,54,57,60,61,62,63,68].

Most studies had observational designs, which limited inference regarding temporal sequence and causality [34,36,37,39,40,41,50]. Longitudinal studies provided stronger evidence regarding the temporal association between early screen exposure and later outcomes [29,31,33,35,38,43,44,45,49,51,70], whereas cross-sectional studies remained vulnerable to reverse causality and residual confounding [32,46,57,64,65,66,67,69]. Experimental studies contributed evidence on short-term effects of specific media content but did not address longer-term developmental outcomes [52,55,56,59].

Because of heterogeneity in study design, screen-exposure measures, neurocognitive outcomes, comparison groups, and reported effect estimates, a quantitative meta-analysis was not conducted. The findings were therefore synthesized narratively by outcome domain and screen-use context.

The design-specific JBI appraisal identified several recurring methodological concerns across the evidence base (Supplementary File S2). Among the 17 cohort studies, the most frequent concern involved exposure measurement, with 14 studies receiving an Unclear judgment regarding the validity and reliability of screen-exposure assessment, largely because exposure was based on caregiver report. Follow-up completeness and handling of missing follow-up data were also incompletely reported in several cohort studies. Among the 22 analytical cross-sectional studies, 18 received an Unclear judgment for exposure measurement, while the cross-sectional nature of these analyses inherently limited temporal inference. Across the four randomized experimental studies, randomization was reported, but allocation concealment and outcome-assessor blinding were absent or insufficiently described in several studies; participant and intervention-provider blinding was generally not feasible because the experimental media conditions were perceptibly different. Overall, neurocognitive outcome measurement and statistical analyses were generally appropriate across study designs. These findings support cautious causal interpretation despite the consistency of associations observed for some outcome domains.

### 3.8. Overall Synthesis Across Neurocognitive Domains

Overall, the consistency of evidence differed across neurocognitive domains. Language development showed the most consistent pattern, particularly for expressive language, vocabulary, and communication outcomes, with less favourable outcomes more frequently associated with higher, passive, solitary, or background screen exposure. However, confidence in these associations was limited by frequent reliance on caregiver-reported exposure measures, variability in language assessment, and potential confounding by socioeconomic and home-learning characteristics. Evidence for general cognitive performance was less consistent, with substantial heterogeneity in developmental constructs, assessment instruments, exposure definitions, and covariate adjustment; several associations were attenuated after adjustment for family-level factors. Attention and self-regulation showed comparatively consistent associations with greater screen exposure, but interpretation was limited by the predominance of observational and cross-sectional designs, parent-reported behavioural measures, and the possibility of reverse causality. Evidence for broader executive functioning was more heterogeneous: associations were clearer for attention and self-regulation than for inhibitory control, working memory, and cognitive flexibility, while experimental studies primarily addressed immediate effects of specific media content rather than long-term developmental outcomes. Taken together, the evidence was therefore most consistent for language and attention-related outcomes, less consistent for general cognition and specific executive-function components, and limited across all domains by methodological heterogeneity, residual confounding, and variation in exposure and outcome measurement.

## 4. Discussion

### 4.1. Principal Findings

This systematic review synthesized evidence from 43 quantitative studies examining associations between screen exposure and neurocognitive outcomes in typically developing children aged 2–5 years. Overall, the findings indicate that higher levels of screen exposure were generally associated with less favorable outcomes in several developmental domains, particularly language development, attention regulation, and executive functioning [30,31,39,40,58,62,63,69,70]. Associations with general cognitive performance were more heterogeneous and appeared to be more sensitive to study design, outcome measurement, and adjustment for family and environmental factors [29,33,38,45,47,51].

The most consistent pattern across the evidence base concerned language development. Higher total screen exposure, passive television viewing, background media exposure, and solitary screen use were commonly associated with poorer expressive language, vocabulary, communication skills, or language-related developmental outcomes [30,31,34,35,39,40,48,51]. In contrast, associations were weaker, inconsistent, or occasionally neutral when media use involved caregiver co-viewing, joint media engagement, or educational content [28,29,39,40,65]. These findings suggest that the developmental relevance of screen exposure may depend not only on duration, but also on the social and cognitive context in which media are used [35,39,40,41].

The present findings should be interpreted in relation to previous meta-analytic evidence. Madigan et al. similarly found less favourable language outcomes with greater screen duration and background television exposure and more favourable associations with educational content and caregiver co-viewing [22]. Bustamante et al., however, found no significant overall association between total screen time and executive functioning in children younger than 6 years, consistent with the greater heterogeneity observed for specific executive-function components in the present review [23]. Mallawaarachchi et al. subsequently demonstrated that screen-use context—including program viewing, background television, and co-use—was associated with cognitive outcomes across children from birth to 5.99 years [24]. The present review therefore does not interpret context sensitivity itself as a novel finding; rather, it extends this literature through a narrower preschool-age focus, an updated evidence base through 2025, and direct comparison of the consistency and methodological limitations of associations across language, cognition, attention, and executive-function domains.

Attention regulation and executive functioning also emerged as important outcome domains. Greater screen exposure was frequently associated with increased inattention, reduced task persistence, poorer self-regulation, and weaker performance on selected executive-function measures [54,57,58,62,63,67,69,70]. However, associations involving working memory, inhibitory control, and cognitive flexibility were less consistent, partly because these outcomes were assessed using diverse instruments and were measured less frequently than language or general developmental outcomes [52,56,59,61,68].

Taken together, the findings support a context-sensitive interpretation of early childhood screen exposure. Rather than functioning as a uniform developmental risk factor, screen use appears to be embedded within broader family routines, caregiving practices, home learning environments, and socioeconomic conditions [31,38,39,40,51,69]. The available evidence therefore does not support a simple interpretation that all forms of screen use have equivalent developmental implications [28,29,37,56,65].

When interpreted alongside the JBI risk-of-bias assessment, however, the consistency of some observed associations should not be equated with high certainty of causal effects. The strongest support was provided by larger prospective studies using standardized neurocognitive outcomes and adjustment for important child, family, and socioeconomic confounders. Nevertheless, frequent reliance on caregiver-reported screen exposure, residual confounding, limited temporal inference in cross-sectional analyses, and incomplete reporting of follow-up or allocation procedures reduced confidence in causal interpretation. These methodological considerations are particularly important because screen exposure is embedded within broader family, socioeconomic, behavioural, and developmental contexts that may independently influence neurocognitive outcomes.

### 4.2. Screen Exposure and Language Development

Language development was the most consistently represented neurocognitive domain in the included studies. Across cross-sectional and longitudinal investigations, greater screen exposure was commonly associated with less favorable expressive language, communication, vocabulary, and language-related developmental outcomes [30,31,34,39,40,45,46,48,51]. This pattern was particularly evident in studies assessing television exposure, mobile-device use, and cumulative daily screen time [30,31,39,46,48].

The findings are consistent with the importance of reciprocal interaction for early language development. Screen use may be associated with fewer opportunities for turn-taking, shared reading, conversation, and caregiver–child engagement when it displaces these activities [28,29,35,39,40]. This interpretation may be particularly relevant for passive or solitary viewing, although the observational nature of much of the evidence precludes causal inference.

The findings concerning expressive language were generally more consistent than those concerning receptive language [39,40,48]. Expressive language requires active verbal practice, word retrieval, sentence production, and responsive interaction [28,29,39,40]. In contrast, receptive language may be influenced by a broader range of environmental inputs, including passive exposure to spoken language [34,39,48]. Nevertheless, passive exposure alone is unlikely to provide the same developmental benefit as interactive, contingent communication with caregivers [28,39,40].

Media context appeared to be particularly relevant. Caregiver co-viewing, joint media engagement, and educational content were associated with weaker adverse or more favourable language-related findings in several studies [28,29,39,40,50]. However, these findings do not demonstrate that educational media or co-viewing eliminate the potential developmental implications associated with prolonged screen exposure.

### 4.3. Screen Exposure and General Cognitive Performance

Associations between screen exposure and general cognitive performance were more variable than those observed for language development [29,33,37,38,41,42,45,47,49]. Several studies reported that greater screen exposure was associated with lower developmental screening scores, weaker problem-solving abilities, reduced communication performance, or lower achievement of developmental milestones [31,33,43,44,45,47,49]. However, the strength of these associations was often reduced after adjustment for socioeconomic status, parental education, home literacy environment, caregiver interaction, and baseline child characteristics [31,38,41,42,49].

This heterogeneity is understandable because general cognitive performance is a broad construct. Included studies assessed cognition using a range of measures, including developmental screening instruments, global developmental indices, memory tasks, problem-solving tasks, early educational skills, and parent-reported developmental outcomes [31,33,36,42,43,45,47]. These measures do not capture identical constructs and may differ considerably in sensitivity, validity, and developmental relevance.

The mixed findings also suggest that total screen time may be an incomplete proxy for developmental experience [29,37,41,65]. Interactive applications, video communication, and age-appropriate educational media may involve different cognitive demands than passive television viewing or background media exposure [29,37,41,65]. Similarly, children who engage with educational content may differ from other children in parental education, home learning resources, caregiver involvement, and access to enrichment opportunities [29,38,41,42]. These family-level differences may partly explain associations between screen exposure and cognitive outcomes [31,38,41,42].

Therefore, the current evidence does not support a uniform conclusion that all screen use is associated with lower general cognitive performance [29,37,41,65]. Instead, it suggests that the relationship is likely context-dependent and influenced by the nature of the media experience, the child’s developmental stage, and the broader home environment [29,37,38,41,42,65].

### 4.4. Screen Exposure, Attention, and Executive Functioning

Executive functioning is a particularly important developmental domain during the preschool years because attention regulation, inhibitory control, working memory, and self-regulation contribute to school readiness, behavioral adjustment, and later academic functioning. Across the included studies, higher screen exposure was commonly associated with more attention-related difficulties, weaker self-regulation, reduced task persistence, and less favorable executive-function outcomes [54,57,58,62,63,67,69,70].

Several observational studies reported associations between greater daily screen exposure and increased inattention or behavioral dysregulation [54,57,63,67,69]. These findings were observed across television viewing, mobile-device use, electronic game play, and broader measures of screen exposure [54,60,63,64,67]. However, because many studies used cross-sectional designs, reverse causality remains possible [57,60,61,64,68]. For example, children with pre-existing attentional difficulties, behavioral dysregulation, or lower self-regulatory capacity may be more likely to receive screens as a calming, distracting, or routine-management strategy.

Experimental studies contributed a different type of evidence. Some studies reported that exposure to fast-paced, fantastical, or highly stimulating screen content was associated with poorer immediate performance on executive-function tasks [52,55,56,59]. These findings are relevant because they suggest that media characteristics may influence short-term attentional engagement and self-regulation. However, short-term experimental effects should not be equated with long-term developmental impairment. Immediate changes in task performance do not establish that repeated exposure causes persistent executive-function deficits.

The evidence regarding inhibitory control, working memory, and cognitive flexibility was less consistent [55,56,59,61,68]. This may reflect both genuine variation in associations and methodological limitations. Executive functions were measured using diverse behavioral tasks, parent-reported questionnaires, and broader behavioral scales [52,53,57,58,67,68,69]. As a result, studies often captured different components of executive functioning, making direct comparison difficult.

### 4.5. Dose, Timing, and Persistence of Screen Exposure

Several included studies suggested that associations between screen exposure and neurocognitive outcomes may become more evident at higher levels of daily use [30,31,39,48,58]. In particular, studies using categorical exposure thresholds frequently reported less favorable outcomes among children exposed to approximately two or more hours of screen time per day [30,58]. However, this pattern should not be interpreted as evidence of a universal biological threshold.

The observed thresholds differed between studies because exposure was measured using different tools and reported in different ways [31,39,48,58]. Some studies examined total daily screen time, whereas others focused on television viewing, mobile-device use, background media, or specific content types [28,31,35,48,56,66]. Therefore, a two-hour threshold may be useful as a descriptive indicator within particular studies, but it should not be interpreted as a precise developmental cutoff applicable to all children and media contexts [30,48,58].

Timing also appeared to be important. Several longitudinal studies suggested that exposure during infancy, toddlerhood, or the early preschool period was associated with later language, communication, cognitive, or attention-related outcomes [28,29,31,33,38,43,44,45,49]. This may reflect the rapid maturation of language and executive-function systems during early childhood. However, early exposure may also be associated with unmeasured family characteristics, including parental stress, limited childcare access, socioeconomic disadvantage, caregiver media habits, and reduced opportunities for shared play or reading [31,35,38,39,40,41].

Persistent exposure may be more relevant than exposure measured at a single point in time [38,51]. Children who experience consistently high levels of screen use across multiple developmental periods may have fewer opportunities for repeated caregiver interaction, structured play, sleep, and language-rich routines [35,39,40,41]. Nevertheless, the available evidence remains insufficient to determine whether cumulative screen exposure has an independent developmental effect after accounting for changing family circumstances and child behavior over time [31,38,51].

### 4.6. Contextual Characteristics of Screen Use

One of the most important findings of this review is that screen exposure should not be considered a single, uniform behavior. The developmental implications of media use appear to vary according to content type, degree of interactivity, caregiver involvement, background exposure, and the broader family environment [28,29,39,40,41,50,65,66].

Passive viewing, entertainment-oriented content, rapidly paced programs, and background television were more commonly associated with less favorable outcomes [28,34,52,55,56,59,66]. Background media may be particularly relevant because it can interrupt play, reduce caregiver-child conversation, fragment joint attention, and alter family routines even when the child is not actively watching [41,42,50,66]. These associations may partly reflect reduced interaction quality rather than a direct effect of screen exposure alone [41,42,50].

Caregiver co-viewing and joint media engagement were generally associated with more favourable language-related outcomes [39,40,50]. The developmental relevance of co-viewing is likely to depend on active caregiver engagement, including conversation and contextualization of media content, rather than simply the caregiver’s physical presence [39,40,42,50].

Family socioeconomic conditions and home learning environments also appear to influence both media use and developmental outcomes [31,38,39,40,41,53,69]. Families experiencing financial strain, time pressure, caregiver stress, limited childcare access, or reduced access to educational resources may rely more heavily on screens as part of daily routines [41,42,53,69]. Consequently, screen exposure may sometimes function as a marker of broader social and environmental disadvantage rather than an isolated causal factor [31,38,40,41,69]. This does not diminish the potential relevance of screen use, but it emphasizes the need for non-stigmatizing and family-centered guidance.

### 4.7. Potential Mechanisms

Several plausible mechanisms may explain the associations identified in this review. The displacement hypothesis remains one of the most relevant [35,39,40,41,42]. Screen use may displace developmentally important activities, including caregiver–child interaction, shared reading, play, physical activity, and sleep, providing one plausible pathway for the observed associations.

A second possible mechanism involves the cognitive and attentional characteristics of screen media. Fast-paced content, frequent scene changes, highly salient audiovisual stimuli, and fragmented narratives may place demands on immature attentional systems [52,55,56,59]. Repeated exposure to such content may be associated with difficulties in sustaining attention, delaying responses, or maintaining engagement in slower-paced activities. However, these mechanisms remain theoretical and require stronger longitudinal and experimental evidence [52,55,56,59].

Sleep may also represent an important pathway [37]. Evening screen use, screen exposure before bedtime, and the presence of screens in children’s bedrooms may be associated with delayed sleep onset, reduced sleep duration, or disrupted sleep routines. Since sleep is closely related to attention, memory consolidation, emotional regulation, and executive functioning, sleep disruption may partially mediate associations between screen exposure and neurocognitive outcomes. Few included studies were designed to evaluate mediation formally, and this remains an important priority for future research.

### 4.8. Strengths and Limitations

This review has several strengths. It focused specifically on children aged 2–5 years, a developmental period characterized by rapid growth in language, cognition, and executive functioning. It also considered multiple neurocognitive domains rather than limiting the synthesis to a single developmental outcome. In addition, the review examined contextual features of screen exposure, including passive versus interactive use, background media, content characteristics, and caregiver co-viewing (Table 2, Figure 2).

However, several limitations should be considered. First, measurement of screen exposure represents a major source of heterogeneity across the evidence base. Studies differed substantially in how exposure was defined and quantified, including total daily screen time, television viewing, mobile-device use, background media, specific content types, and duration-based thresholds. Consequently, apparently similar exposure categories may represent substantially different media experiences, limiting comparability across studies and preventing identification of a uniform exposure–outcome relationship. Most studies also relied predominantly on parent- or caregiver-reported estimates of screen use. Such measures are vulnerable to recall and social-desirability bias and may provide only approximate estimates of daily exposure, particularly when children use multiple devices or when background media, media content, caregiver co-viewing, and concurrent activities are not captured. Measurement error may therefore contribute to both attenuation and variability of observed associations. More objective approaches, including device-generated usage data and repeated time-use measures, would improve exposure characterization in future studies.

Second, neurocognitive outcomes were assessed using diverse instruments. Language outcomes included standardized tests, vocabulary measures, communication scales, and parent-reported assessments. Executive functioning was measured using behavioral tasks, parent questionnaires, attention scales, and broader self-regulation measures. This heterogeneity limited direct comparison between studies and prevented quantitative meta-analysis.

Third, most included studies were observational. Although longitudinal studies provided stronger evidence regarding temporal sequence, residual confounding and reverse causality could not be excluded. Screen exposure is closely connected with socioeconomic conditions, parental education, family routines, caregiver stress, home literacy environment, sleep, and child temperament. Even well-adjusted observational studies may not fully account for these interconnected influences.

Fourth, the review included only peer-reviewed English-language publications identified through PubMed, Web of Science, and Scopus and excluded grey literature and unpublished studies. Consequently, publication and language bias cannot be excluded, as studies reporting null or less pronounced associations may be less likely to appear in the peer-reviewed literature captured by the search strategy. Relevant evidence published in other languages, indexed in additional databases, or available in unpublished formats may therefore not have been identified. Formal statistical assessment of publication bias was not considered appropriate because substantial heterogeneity in study designs, exposure definitions, neurocognitive outcomes, and reported effect estimates precluded quantitative meta-analysis. The potential influence of publication bias should therefore be considered when interpreting the overall consistency of the evidence.

Fifth, a methodological limitation concerns the timing of PROSPERO registration. The review was registered after the formal literature search and study-selection process had been completed and therefore cannot be considered prospectively registered. Prospective registration is important because it documents the intended eligibility criteria, outcomes, and synthesis methods before the review process is completed, thereby increasing methodological transparency and reducing the possibility of post hoc methodological decisions or selective reporting. Consequently, retrospective registration cannot provide the same level of assurance that all methodological decisions were specified independently of the emerging evidence. To improve transparency, the timing of registration has been explicitly reported in the Methods, and deviations between the registered methods and the final review are documented in Supplementary Table S1. Nevertheless, the absence of prospective registration should be considered when interpreting the methodological robustness of the review.

Finally, although the use of design-specific JBI critical appraisal tools provided a standardized assessment of methodological quality and risk of bias, several recurring methodological concerns remained across the evidence base. These included uncertainty regarding the validity of screen-exposure measurement, incomplete reporting of attrition in some cohort studies, limited temporal inference in observational studies, and insufficient reporting of allocation procedures or blinding in some experimental studies. These limitations reduce the certainty of causal conclusions despite relatively consistent associations in selected neurocognitive domains (Supplementary File S2).

### 4.9. Implications for Clinical Practice and Public Health

The findings support a nuanced approach to early childhood media guidance. Recommendations for families should not focus exclusively on total screen time. Clinicians, educators, and public-health professionals should also consider the type of content, the timing of media use, whether screens are used during meals or before bedtime, the presence of background television, and the extent of caregiver involvement [35,37,39,40,66].

Practical advice may include reducing passive and background media exposure, avoiding screens as the primary strategy for behavior regulation, protecting sleep and screen-free family routines, encouraging shared reading and unstructured play, and promoting caregiver co-viewing when screen use occurs. Guidance should be supportive rather than judgmental, particularly for families facing socioeconomic stress, limited childcare options, or high caregiving demands.

At a population level, interventions should emphasize media literacy, family media planning, access to high-quality early childhood education, caregiver support, and equitable opportunities for language-rich play and interaction. Screen exposure should be viewed as one potentially modifiable element within a broader developmental environment.

### 4.10. Directions for Future Research

Future research should move beyond broad estimates of total daily screen time. More precise exposure measurement is needed, including device-generated usage data, time-use diaries, repeated assessments of media context, content classification, and measures of caregiver co-viewing. Studies should distinguish between foreground and background exposure, passive and interactive use, educational and entertainment content, and individual versus shared media experiences.

Prospective longitudinal studies should assess screen exposure and neurocognitive development repeatedly across early childhood. Such studies should measure baseline developmental characteristics, family socioeconomic conditions, caregiver mental health, parenting practices, home literacy environment, childcare attendance, sleep, physical activity, and child temperament. Analytical approaches that strengthen causal inference, including propensity-score methods, sibling-comparison designs, fixed-effects models, and mediation analyses, may help clarify whether observed associations are attributable to screen exposure itself or to broader family and environmental factors.

Well-designed intervention studies are also needed. Pragmatic trials could examine whether reducing background television, limiting passive viewing, improving caregiver co-viewing, introducing screen-free routines, or replacing screen time with shared reading and play leads to measurable improvements in language, attention, or self-regulation. Such studies should assess both feasibility and equity, ensuring that recommendations are realistic for families with different levels of time, resources, and support.

## 5. Conclusions

This systematic review of 43 quantitative studies found that greater screen exposure in typically developing children aged 2–5 years was generally associated with less favorable neurocognitive outcomes, particularly in language development, attention regulation, and aspects of executive functioning. Associations with general cognitive performance were less consistent and appeared to depend more strongly on study design, exposure measurement, developmental assessment, and adjustment for family and environmental factors.

The findings indicate that screen exposure should not be considered a uniform developmental exposure. Passive viewing, background media, prolonged daily use, entertainment-oriented or highly stimulating content, and solitary screen use were more frequently associated with unfavorable outcomes. In contrast, caregiver co-viewing, joint media engagement, and developmentally appropriate content were associated with weaker adverse associations or more neutral findings in several studies. These observations highlight the importance of considering not only screen duration, but also media content, timing, context of use, and the quality of caregiver–child interaction.

However, the evidence base remains limited by substantial heterogeneity in exposure definitions, neurocognitive outcome measures, and study designs. Most included studies relied on parent-reported screen-use estimates and observational designs, meaning that residual confounding and reverse causality cannot be excluded. Accordingly, the findings should be interpreted as evidence of associations rather than evidence that screen exposure independently causes adverse neurocognitive outcomes. Screen exposure is closely intertwined with socioeconomic conditions, home learning environment, caregiver stress, parenting practices, sleep, and child temperament.

Accordingly, the available evidence supports cautious, context-sensitive guidance rather than a one-dimensional focus on screen-time duration alone. Strategies that reduce passive and background media exposure, protect sleep and screen-free family routines, encourage shared reading and unstructured play, and promote active caregiver involvement when screens are used may be particularly relevant during the preschool period. Future longitudinal studies and pragmatic interventions using standardized developmental assessments and more precise measures of media exposure are needed to clarify causal pathways and identify developmentally supportive patterns of digital-media use.

## Figures and Tables

**Table 1:** Overview of studies included in the systematic review (*n* = 43).

Study	Design	Participant Period/Developmental Stage	Screen Exposure Assessed	Primary Neurocognitive Outcome(s)	Main Finding Included in the Narrative Synthesis
Linebarger and Walker, 2005 [28]	Longitudinal observational study	Infancy to toddlerhood; outcomes at approximately 30 months	Television viewing logs; programme content and intended audience	Vocabulary; expressive language	Associations varied substantially by programme type; several child-directed programmes were associated with more favourable language scores, whereas others were associated with less favourable outcomes.
Barr et al., 2010 [29]	Longitudinal observational study	Infancy/early childhood; cognitive outcomes at 4 years	Adult-directed and child-directed television exposure	Cognitive skills; school readiness	Television content and intended audience showed differential associations with cognitive skills at age 4 years.
Duch et al., 2013 [30]	Cross-sectional and longitudinal study	Hispanic infants and toddlers; 1-year follow-up	Duration and content of screen-media exposure	Communication and language development	Television viewing exceeding 2 h/day was associated with increased odds of low communication scores.
Madigan et al., 2019 [31]	Longitudinal cohort study	Children assessed from 24 to 60 months	Parent-reported total screen time	Developmental screening outcomes	Higher screen time at earlier assessments was associated with lower later developmental-screening scores.
Hutton et al., 2020 [32]	Cross-sectional neuroimaging study	Preschool-aged children	Screen-based media use measured using a structured parent questionnaire	Emergent literacy; language-related white-matter integrity	Greater screen-media use was associated with lower integrity of white-matter tracts supporting language and emergent literacy.
Supanitayanon et al., 2020 [33]	Longitudinal cohort study	Screen exposure during the first 2 years; preschool cognitive outcomes	Early screen-media exposure	Preschool cognitive development	Greater early screen exposure was associated with less favourable preschool cognitive development.
Sundqvist et al., 2021 [34]	Observational study	Children at approximately 2 years of age	Digital-media exposure and media-use context	Language development	Higher levels of digital-media exposure were associated with less favourable language outcomes in some analyses.
Martinot et al., 2021 [35]	Longitudinal cohort study	Children assessed at ages 2, 3, and 5–6 years	Total screen time and television exposure during family meals	Language development; verbal IQ	Greater exposure to television during family meals was associated with less favourable later language outcomes, whereas associations with overall screen time were more heterogeneous after adjustment for family and child characteristics.
John et al., 2021 [36]	Cross-sectional study	Preschool children	Parent-reported screen time	Parent-reported cognitive delay	Greater screen exposure was associated with a higher likelihood of parent-reported cognitive delay.
Axelsson et al., 2022 [37]	Observational study	Preschool-aged children	Engagement with screen content	Cognitive development; sleep	Associations between screen-content engagement and cognitive development varied according to media-use characteristics and sleep-related factors.
McArthur et al., 2022 [38]	Longitudinal cohort study	Preschool children	Screen time across early childhood	Developmental and behavioural outcomes	Greater screen exposure was associated with less favourable developmental and behavioural outcomes in several analyses.
Mustonen et al., 2022 [39]	Cross-sectional study	Children aged approximately 2.5–4 years	Child and maternal screen time; co-viewing	Multiple language domains	Higher child screen time was associated with less favourable language development, whereas associations differed by screen-use context.
Medawar et al., 2023 [40]	Cross-sectional study	Toddlers	Screen exposure; home literacy; joint media engagement	Early language outcomes	Home literacy and joint media engagement were associated with more favourable language outcomes; higher screen exposure showed less favourable associations.
Dore et al., 2023 [41]	Time-diary observational study	Preschool-aged children	Home media use and daily media routines	School-readiness and early learning skills	Associations varied according to the type of home media activity and its place within daily routines.
Rai et al., 2023 [42]	Pilot observational study	Preschool-aged children	Patterns of screen time and parent–child interaction	Cognitive development	Screen-use patterns were examined in relation to parent–child interaction and cognitive development; findings were exploratory.
Yamamoto et al., 2023 [43]	Longitudinal population-based cohort study	Children assessed between 1 and 3 years	Screen time	Developmental performance	Greater screen exposure was associated with less favourable developmental performance in early childhood.
Kracht et al., 2023 [44]	Longitudinal study	Infancy through early childhood	Maternal and child screen time	Developmental outcomes	Higher maternal and child screen exposure was examined in relation to less favourable child developmental indicators.
Takahashi et al., 2023 [45]	Longitudinal birth-cohort study	Screen exposure at 1 year; outcomes at 2 and 4 years	Screen time at age 1 year	Communication and problem-solving delay	Greater screen time at age 1 year was associated with later communication and problem-solving developmental delays.
Monteiro et al., 2024 [46]	Cross-sectional study	Preschool-aged children	Digital-media use	Expressive language	Greater digital-media use was associated with lower expressive-language skills.
Binet et al., 2024 [47]	Prospective cohort study	Preschool-aged children during and after the COVID-19 pandemic	Screen time	Achievement of developmental milestones	Greater screen time was prospectively associated with lower achievement of developmental milestones.
Rayce et al., 2024 [48]	Large-scale cross-sectional survey	Toddlers	Mobile-device screen time	Expressive and receptive language	Mobile-device exposure of 1 h/day or more was associated with poorer language-development scores.
Yang et al., 2024 [49]	Longitudinal birth-cohort study	Early childhood	Screen use	Cognitive development	Greater screen use was associated with less favourable cognitive-development outcomes on selected measures.
Gago-Galvagno et al., 2024 [50]	Cross-sectional study	Toddlers	Screen use; dyadic interaction context	Communication and regulation skills	Screen use was associated with communication and regulation outcomes, with dyadic interaction characteristics also contributing.
Gath et al., 2025 [51]	Longitudinal cohort study	Early childhood through later preschool assessment	Screen-time exposure trajectories	Language; early educational skills; peer social functioning	Higher levels of early screen exposure were associated with less favourable later language and educational outcomes.
Lillard and Peterson, 2011 [52]	Experimental study	Preschool-aged children	Short-term exposure to different television content	Executive function	Immediate executive-function performance differed after exposure to fast-paced television content.
Linebarger et al., 2014 [53]	Cross-sectional observational study	Preschool-aged children	Parenting practices, media use, and cumulative risk	Executive functioning	Media use was examined alongside parenting and cumulative risk as correlates of executive functioning.
Nathanson et al., 2014 [54]	Observational study	Preschool-aged children	Television exposure	Executive function	Greater television exposure was associated with less favourable executive-function performance.
Lillard et al., 2015 [55]	Experimental study	Preschool-aged children	Short-term television-content exposure	Executive function	Executive-function findings differed across television-content conditions.
Huber et al., 2018 [56]	Experimental study	Young children	Screen-media content	Executive functioning	Executive-function performance differed according to the characteristics of screen-media content.
Munzer et al., 2018 [57]	Cross-sectional study	Low-income preschool-aged children	Media exposure	Self-regulatory behaviour	Greater media exposure was associated with less favourable self-regulatory behaviour across several measures.
Tamana et al., 2019 [58]	Longitudinal birth-cohort study	Preschool children	Daily screen time	Inattention problems	Greater screen exposure was associated with more inattention-related problems.
Li et al., 2020 [59]	Experimental study	Chinese preschool-aged children	Exposure to fantastical animated television events	Executive function	Exposure to fantastical animated content was associated with differences in immediate executive-function performance.
Yang et al., 2020 [60]	Cross-sectional study	Preschool-aged children	Electronic-game play	Executive functioning	Greater electronic-game use was associated with less favourable executive-function outcomes.
Jusienė et al., 2020 [61]	Cross-sectional study	Preschool-aged children	Screen-based media use	Executive functioning	Associations between screen-media use and executive functioning varied according to type and context of use.
McHarg et al., 2020 [62]	Longitudinal study	Toddlerhood and preschool period	Screen time	Executive function	Greater screen exposure was associated with less favourable executive-function outcomes over time.
Corkin et al., 2021 [63]	Observational study	Preschool-aged children	Screen-media exposure	Executive function; inattention/hyperactivity	Greater screen-media exposure was associated with executive-function and attention-related difficulties.
Konok et al., 2021 [64]	Observational study	Preschool-aged children	Mobile-device use	Attention; socio-cognitive skills	Mobile-device use was associated with local attentional precedence and less favourable socio-cognitive skills.
McNeill et al., 2021 [65]	Cross-sectional study	Preschool-aged children	Application use and media-program viewing	Cognitive and psychosocial development	Associations differed according to whether children used applications or watched media programmes.
Nichols, 2022 [66]	Observational study	Young children	Background television exposure	Executive functioning	Background television exposure was examined as a contextual correlate of executive-function outcomes.
Choe et al., 2022 [67]	Observational study	Young children	Child and parent screen-media use by device type	Self-regulation	Device-specific child and parent media use were associated with child dysregulation and self-regulatory outcomes.
Portugal et al., 2023 [68]	Cross-sectional study	Preschool-aged children	Touchscreen use	Executive function	Executive-function differences were examined among children with higher touchscreen use; findings were mixed across domains.
Horowitz-Kraus et al., 2024 [69]	Cross-sectional EEG study	Preschool-aged children	Parent-reported screen time	Executive function	Greater screen time was negatively associated with executive-function abilities, alongside parental executive function and home literacy factors.
Fitzpatrick et al., 2025 [70]	Longitudinal trajectory study	Preschool-aged children	Screen-time trajectories	Executive function; effortful control	Higher or persistently elevated screen-time trajectories were associated with less favourable executive-function outcomes.

**Table 2:** Narrative synthesis of associations between screen exposure and neurocognitive outcomes in children aged 2–5 years.

Neurocognitive Outcome Domain	Number of Studies *	Main Screen-Exposure Measures	Overall Pattern of Findings	Contextual Characteristics Associated with Findings	Main Interpretation Limitations
Language development	30	Total daily screen time; television viewing; mobile-device and tablet use; background television; screen-use duration and frequency	Higher screen exposure was generally associated with less favourable expressive language, vocabulary, communication skills, and language-related developmental outcomes. Associations were more consistent for expressive than receptive language.	Passive television viewing, solitary use, background media exposure, and prolonged daily use were more frequently associated with less favourable language outcomes. Caregiver co-viewing, joint media engagement, educational content, and a richer home literacy environment were associated with weaker adverse or more neutral findings in several studies.	Predominantly parent-reported screen exposure; variation in language measures; potential confounding by parental education, socioeconomic status, home literacy practices, caregiver interaction, and pre-existing language difficulties.
General cognitive performance	18	Total screen time; television exposure; mobile/tablet use; engagement with digital content; developmental exposure trajectories	Findings were mixed. Several studies reported associations between greater screen exposure and lower developmental-screening scores, poorer communication or problem-solving performance, or lower achievement of developmental milestones. Other associations were attenuated or non-significant after covariate adjustment.	Interactive or developmentally appropriate educational media showed neutral or occasionally more favourable associations in some studies. Passive viewing and background media were more often linked to reduced engagement or less favourable developmental outcomes.	Considerable heterogeneity in cognitive constructs, instruments, timing of outcome assessment, and covariate adjustment. General cognitive measures often combined multiple developmental domains.
Attention regulation and self-regulation	Subset of 16 executive-function studies	Total daily screen time; television exposure; mobile-device use; electronic-game play; background media	Greater screen exposure was frequently associated with increased inattention, distractibility, reduced task persistence, and less favourable self-regulatory behaviour. Associations were reported in both cross-sectional and longitudinal studies.	Fast-paced or highly stimulating content, background media, and passive viewing were more often associated with attention-related difficulties. Family routines, sleep, caregiver involvement, and parent media use were relevant contextual variables in several studies.	Reverse causality remains possible because children with pre-existing attentional or behavioural difficulties may be more likely to receive screens for calming or routine-management purposes. Many studies relied on parent-report behavioural measures.
Executive functioning	16	Television viewing; screen-media content; touchscreen use; mobile-device use; electronic-game play; total screen time	Greater screen exposure was commonly associated with less favourable executive-function outcomes, particularly in relation to self-regulation and selected attention-related measures. Findings for inhibitory control, working memory, and cognitive flexibility were more heterogeneous.	Experimental studies suggested that executive-function performance may differ following exposure to fast-paced or fantastical content. Interactive use and caregiver-mediated media experiences showed more variable or neutral associations.	Executive functioning was measured using diverse parent-reported scales and behavioural tasks, limiting direct comparison. Experimental studies primarily assessed immediate task performance rather than long-term developmental outcomes.
Multiple neurocognitive domains	14	Broad measures of screen time, television viewing, mobile-device use, media content, and caregiver co-viewing	Studies assessing multiple outcomes generally suggested that language and attention-related outcomes showed clearer associations with screen exposure than broad cognitive measures. Evidence did not support a single, uniform effect across all domains.	Associations differed according to content, passive versus interactive use, background media exposure, caregiver co-viewing, family routines, and home learning environment.	Overlap between outcomes, exposure measures, and cohorts limited comparison across studies. The extent to which contextual factors represent confounders, moderators, or mediators was rarely tested formally.

* Outcome categories are not mutually exclusive because several included studies assessed more than one neurocognitive domain. The 14 studies assessing multiple domains are included within the counts for language development, general cognition, and/or executive functioning.

## Data Availability

No new data were created or analyzed in this study. Data sharing is not applicable to this article.

## Supplementary Materials

The following supporting information can be downloaded at: https://www.mdpi.com/article/10.3390/healthcare14183081/s1, File S1: PRISMA 2020 Checklist; File S2: JBI Risk of Bias Assessment; Table S1: Deviations from the PROSPERO Registration Record; Table S2: Complete Database-Specific Search Strategies; Table S3: Study-Level Quantitative Findings of the Included Studies. Reference [71] is cited in the Supplementary Materials.

## Abbreviations

The following abbreviations are used in this manuscript:

PRISMA	Preferred Reporting Items for Systematic Reviews and Meta-Analyses
