# Supplementary material for: Associations Between Early Screen Exposure and Neurocognitive Outcomes in Children Aged 2–5 Years: A Systematic Review
Source: Healthcare (Basel). 2026 Sep 19;14(18):3081. doi: 10.3390/healthcare14183081 (PMC13606092; doi:10.3390/healthcare14183081)
Supplement: Supplementary file 1 [file healthcare-14-03081-s001.zip › Supplementary File S1. PRISMA 2020 Checklist.pdf]

PRISMA 2020 Checklist

Section and Topic	Item #	Checklist item	Location where item is reported
**TITLE**			
Title	1	Identify the report as a systematic review.	p. 1, lines 2–3, Title
**ABSTRACT**			
Abstract	2	See the PRISMA 2020 for Abstracts checklist.	p. 2, lines 20–54, Structured Abstract
**INTRODUCTION**			
Rationale	3	Describe the rationale for the review in the context of existing knowledge.	pp. 3–5, lines 55–150, Sections 1.1–1.4
Objectives	4	Provide an explicit statement of the objective(s) or question(s) the review addresses.	p. 5, lines 151–176, Section 1.5, Aim and Scope of the Present Review
**METHODS**			
Eligibility criteria	5	Specify the inclusion and exclusion criteria for the review and how studies were grouped for the syntheses.	pp. 5–6, lines 177–247, Sections 2.2.1–2.2.5; p. 8, Section 2.7
Information sources	6	Specify all databases, registers, websites, organisations, reference lists and other sources searched or consulted to identify studies. Specify the date when each source was last searched or consulted.	pp. 6–7, lines 239–263, Section 2.3, Information Sources and Search Strategy; final search date reported as 24 June 2026
Search strategy	7	Present the full search strategies for all databases, registers and websites, including any filters and limits used.	pp. 6–7, Section 2.3, Information Sources and Search Strategy. Search concepts, key terms, databases, date limits, and the final search date are reported.
Selection process	8	Specify the methods used to decide whether a study met the inclusion criteria of the review, including how many reviewers screened each record and each report retrieved, whether they worked independently, and if applicable, details of automation tools used in the process.	p. 7, Section 2.4, Study Selection. Title/abstract screening, full-text assessment, consensus resolution of uncertainties, and the absence of automation tools are reported.
Data collection process	9	Specify the methods used to collect data from reports, including how many reviewers collected data from each report, whether they worked independently, any processes for obtaining or confirming data from study investigators, and if applicable, details of automation tools used in the process.	pp. 7–8, Section 2.5, Data Extraction. A standardized extraction framework and extracted variables are described.
Data items	10a	List and define all outcomes for which data were sought. Specify whether all results that were compatible with each outcome domain in each study were sought (e.g. for all measures, time points, analyses), and if not, the methods used to decide which	pp. 6–8, Section 2.2.4, Outcomes, and Section

PRISMA 2020 Checklist

Section and Topic	Item #	Checklist item	Location where item is reported
		results to collect.	2.5, Data Extraction.
	10b	List and define all other variables for which data were sought (e.g. participant and intervention characteristics, funding sources). Describe any assumptions made about any missing or unclear information.	pp. 7–8, Section 2.5, Data Extraction. Study characteristics, exposure measures, outcome measures, covariates, reported effect estimates, and study limitations were extracted where available.
Study risk of bias assessment	11	Specify the methods used to assess risk of bias in the included studies, including details of the tool(s) used, how many reviewers assessed each study and whether they worked independently, and if applicable, details of automation tools used in the process.	p. 8, Section 2.6, Methodological Quality Considerations. Methodological quality was assessed narratively according to exposure measurement, outcome assessment, study design, temporal sequence, confounding adjustment, sample size, retention, and potential sources of bias.
Effect measures	12	Specify for each outcome the effect measure(s) (e.g. risk ratio, mean difference) used in the synthesis or presentation of results.	pp. 7–8, Sections 2.5 and 2.7. Study-specific reported effect estimates were extracted where available and interpreted narratively; no common effect measure or pooled estimate was used because of substantial heterogeneity.
Synthesis methods	13a	Describe the processes used to decide which studies were eligible for each synthesis (e.g. tabulating the study intervention characteristics and comparing against the planned groups for each synthesis (item #5)).	pp. 5–8, Sections 2.2 and 2.7.
	13b	Describe any methods required to prepare the data for presentation or synthesis, such as handling of missing summary statistics, or data conversions.	Not applicable—no quantitative pooling, data conversion, imputation, or transformation of summary estimates was undertaken because the evidence was synthesized narratively; see Section 2.7, p. 8.
	13c	Describe any methods used to tabulate or visually display results of individual studies and syntheses.	Figure 1, p. 9; Table 1, pp. 10–18; Table 2, pp. 20–

Section and Topic	Item #	Checklist item	Location where item is reported
			23; Figure 2, p. 23.
	13d	Describe any methods used to synthesize results and provide a rationale for the choice(s). If meta-analysis was performed, describe the model(s), method(s) to identify the presence and extent of statistical heterogeneity, and software package(s) used.	p. 8, lines 322–337, Section 2.7, Data Synthesis.
	13e	Describe any methods used to explore possible causes of heterogeneity among study results (e.g. subgroup analysis, meta-regression).	p. 8, Section 2.7; Sections 3.6–3.8, pp. 19–24; Table 2, pp. 20–23.
	13f	Describe any sensitivity analyses conducted to assess robustness of the synthesized results.	Not applicable—no sensitivity analyses were conducted because no quantitative synthesis or meta-analysis was performed; see Section 2.7, p. 8.
Reporting bias assessment	14	Describe any methods used to assess risk of bias due to missing results in a synthesis (arising from reporting biases).	Partially reported: p. 8, Section 2.6, Methodological Quality Considerations. Potential reporting bias was considered within the narrative methodological appraisal; no formal synthesis-level assessment of bias due to missing results was undertaken.
Certainty assessment	15	Describe any methods used to assess certainty (or confidence) in the body of evidence for an outcome.	p. 8, lines 337–345, Section 2.8, Assessment of Evidence Strength. Evidence strength was interpreted narratively.
**RESULTS**			
Study selection	16a	Describe the results of the search and selection process, from the number of records identified in the search to the number of studies included in the review, ideally using a flow diagram.	p. 9, lines 346–367, Section 3.1, Study Selection; Figure 1, p. 9.
	16b	Cite studies that might appear to meet the inclusion criteria, but which were excluded, and explain why they were excluded.	p. 9, Section 3.1, Study Selection, and Figure 1. Full-text exclusion categories and their respective counts are reported.
Study characteristics	17	Cite each included study and present its characteristics.	Section 3.2, pp. 10–18; Table 1, pp. 10–18.

PRISMA

PRISMA 2020 Checklist

Section and Topic	Item #	Checklist item	Location where item is reported
Risk of bias in studies	18	Present assessments of risk of bias for each included study.	Partially reported: p. 8, Section 2.6, Methodological Quality Considerations. Narrative methodological considerations were incorporated into the synthesis; individual study-level risk-of-bias judgments were not presented.
Results of individual studies	19	For all outcomes, present, for each study: (a) summary statistics for each group (where appropriate) and (b) an effect estimate and its precision (e.g. confidence/credible interval), ideally using structured tables or plots.	Partially reported: Table 1, pp. 10–18, and Sections 3.3–3.7, pp. 18–20. Study characteristics and main findings were presented narratively; standardized effect estimates and precision measures were not consistently reported because of heterogeneity across studies.
Results of syntheses	20a	For each synthesis, briefly summarise the characteristics and risk of bias among contributing studies.	Partially reported: Table 2, pp. 20–23, and Section 3.8, pp. 23–24. Study characteristics, methodological heterogeneity, and major interpretive limitations were summarized; formal study-level risk-of-bias ratings were not presented.
	20b	Present results of all statistical syntheses conducted. If meta-analysis was done, present for each the summary estimate and its precision (e.g. confidence/credible interval) and measures of statistical heterogeneity. If comparing groups, describe the direction of the effect.	Not applicable—no statistical synthesis or meta-analysis was conducted; findings were synthesized narratively by outcome domain and screen-use context; see Section 2.7, p. 8, and Section 3.8, p. 24.
	20c	Present results of all investigations of possible causes of heterogeneity among study results.	Sections 3.6–3.8, pp. 19–24; Table 2, pp. 20–23.
	20d	Present results of all sensitivity analyses conducted to assess the robustness of the synthesized results.	Not applicable—no

PRISMA 2020 Checklist

Section and Topic	Item #	Checklist item	Location where item is reported
			sensitivity analyses were conducted because no quantitative synthesis or meta-analysis was performed.
Reporting biases	21	Present assessments of risk of bias due to missing results (arising from reporting biases) for each synthesis assessed.	Partially reported: p. 8, Section 2.6, Methodological Quality Considerations. Potential reporting bias was considered narratively; no formal assessment of bias due to missing results was undertaken.
Certainty of evidence	22	Present assessments of certainty (or confidence) in the body of evidence for each outcome assessed.	p. 8, Section 2.8, Assessment of Evidence Strength; Table 2, pp. 20–23. The overall strength of evidence was interpreted narratively, with greater weight given to longitudinal designs, standardized assessments, larger samples, and adjusted analyses.
**DISCUSSION**			
Discussion	23a	Provide a general interpretation of the results in the context of other evidence.	Sections 4.1–4.7, pp. 24–29, lines 556–753.
	23b	Discuss any limitations of the evidence included in the review.	p. 29, lines 754–802, Section 4.8, Strengths and Limitations.
	23c	Discuss any limitations of the review processes used.	p. 29, lines 754–802, Section 4.8, Strengths and Limitations.
	23d	Discuss implications of the results for practice, policy, and future research.	Sections 4.9–4.10, pp. 29–30, lines 803–852.
**OTHER INFORMATION**			
Registration and protocol	24a	Provide registration information for the review, including register name and registration number, or state that the review was not registered.	p. 5, Section 2.1, Review Design and Reporting Framework: PROSPERO registration number CRD420261426450.
	24b	Indicate where the review protocol can be accessed, or state that a protocol was not prepared.	p. 5, Section 2.1, Review

PRISMA 2020 Checklist

Section and Topic	Item #	Checklist item	Location where item is reported
			Design and Reporting Framework: a separate full protocol was not published; PROSPERO record CRD420261426450 is publicly available.
	24c	Describe and explain any amendments to information provided at registration or in the protocol.	p. 5, Section 2.1, Review Design and Reporting Framework; Supplementary Table S1.
Support	25	Describe sources of financial or non-financial support for the review, and the role of the funders or sponsors in the review.	p. 31, lines 884–885, Funding statement.
Competing interests	26	Declare any competing interests of review authors.	p. 31, lines 894–895, Conflicts of Interest statement.
Availability of data, code and other materials	27	Report which of the following are publicly available and where they can be found: template data collection forms; data extracted from included studies; data used for all analyses; analytic code; any other materials used in the review.	p. 31, lines 890–893, Data Availability Statement.

From: Page MJ, McKenzie JE, Bossuyt PM, Boutron I, Hoffmann TC, Mulrow CD, et al. The PRISMA 2020 statement: an updated guideline for reporting systematic reviews. BMJ 2021;372:n71. doi: 10.1136/bmj.n71.
This work is licensed under CC BY 4.0. To view a copy of this license, visit https://creativecommons.org/licenses/by/4.0/
