# Supplementary material for: Associations Between Early Screen Exposure and Neurocognitive Outcomes in Children Aged 2–5 Years: A Systematic Review
Source: Healthcare (Basel). 2026 Sep 19;14(18):3081. doi: 10.3390/healthcare14183081 (PMC13606092; doi:10.3390/healthcare14183081)
Supplement: Supplementary file 1 [file healthcare-14-03081-s001.zip › Supplementary Table S1. Deviations from the PROSPERO Registration Record.pdf]

Supplementary Table S1. Deviations from the PROSPERO Registration Record

Registered PROSPERO method	Final review method	Reason for deviation
The PROSPERO record specified searches in CINAHL, ERIC, PsycINFO, PubMed, SCIE, SSCI, and Scopus.	The final review searched PubMed, Web of Science, and Scopus.	The final search strategy was restricted to three major biomedical and multidisciplinary databases for which complete, reproducible searches could be conducted and documented. Web of Science was used as the multidisciplinary citation-index database in the final search strategy.
The PROSPERO record planned backward reference-list checking and forward citation searching to identify additional eligible studies.	The final review used a database-only identification strategy, and no additional studies were included through citation searching or reference-list screening.	The final review adopted a clearly reproducible database-based search approach. The PRISMA 2020 flow diagram therefore reports identification through databases only.
The PROSPERO record did not specify an upper publication-date limit after 1 January 2000.	The final review included studies published between 1 January 2000 and 31 December 2025.	A fixed end date of 31 December 2025 was applied to define a reproducible and stable evidence base before final screening, data extraction, and narrative synthesis.
The PROSPERO record focused on children aged 2–5 years and described eligible outcome assessments as occurring during the preschool period or follow-up assessments linked to screen exposure measured between ages 2 and 5 years.	The final review included several longitudinal studies in which screen exposure was measured during infancy or toddlerhood, provided that relevant neurocognitive outcomes were assessed between ages 2 and 5 years.	This clarification was introduced to capture longitudinal evidence on developmental timing of screen exposure while maintaining the prespecified target age range for neurocognitive outcomes.
The PROSPERO record specified formal risk-of-bias assessment using design-specific tools, including RoB 2, JBI tools, ROBINS-E, and ROBINS-I.	Methodological quality was evaluated narratively, with consideration of exposure measurement, outcome assessment, study design, adjustment for confounding, temporal sequence, sample size, attrition, and potential sources of bias.	The final evidence base included highly heterogeneous observational and experimental designs, exposure definitions, and outcome measures. A narrative methodological appraisal was therefore used to support a context-sensitive narrative synthesis.

The PROSPERO record planned assessment of reporting bias by comparison with protocols or registrations where available and consideration of funnel plots and Egger's test when meta-analysis was feasible.	No formal reporting-bias assessment was undertaken.	Meta-analysis was not feasible because of substantial heterogeneity in populations, exposure measures, outcome definitions, study designs, and reported effect estimates. Protocols and registrations were not consistently available for the included studies.
The PROSPERO record planned GRADE assessment of certainty for the main outcome domains.	The overall strength of evidence was interpreted narratively, without formal GRADE ratings.	The heterogeneity of study designs, exposure measurements, outcome instruments, and effect estimates precluded consistent outcome-specific GRADE ratings. Evidence strength was therefore considered narratively, giving greater weight to longitudinal designs, standardized assessments, larger samples, and adjusted analyses.
The PROSPERO record stated that meta-analysis would be considered where studies were sufficiently homogeneous.	No meta-analysis was performed; findings were synthesized narratively by outcome domain and screen-use context.	This was not a methodological deviation. Narrative synthesis was prespecified as the alternative approach when meta-analysis was not feasible. The included studies were insufficiently comparable in population characteristics, exposure definitions, outcome measures, study designs, and reported effect estimates.
