# Supplementary material for: Associations Between Early Screen Exposure and Neurocognitive Outcomes in Children Aged 2–5 Years: A Systematic Review
Source: Healthcare (Basel). 2026 Sep 19;14(18):3081. doi: 10.3390/healthcare14183081 (PMC13606092; doi:10.3390/healthcare14183081)
Supplement: Supplementary file 1 [file healthcare-14-03081-s001.zip › Supplementary_File_S2_JBI_Risk_of_Bias_Assessment 2.pdf]

SUPPLEMENTARY FILE S2

JBI design-specific risk-of-bias assessment of the 43 studies included in the systematic review

Assessment framework. Risk of bias was assessed with the current design-specific JBI critical appraisal tools appropriate to the analytical component contributing to this review: the revised JBI tool for analytical cross-sectional studies (2026), the revised JBI tool for cohort studies (2025), and the revised JBI tool for randomized controlled trials (2023).

Study-design assignment. Tool selection was based on the analytical component that contributed the neurocognitive association to the present synthesis, rather than solely on the parent study label. Mixed-design reports were therefore appraised according to the relevant component. In particular, Duch et al. [30] was appraised as a cohort study for its longitudinal component; McArthur et al. [38] and Tamana et al. [58] were appraised as analytical cross-sectional studies for the principal concurrent associations summarized in this review; Konok et al. [64] was appraised for the observational analytical cross-sectional component; and Portugal et al. [68] was appraised as a cohort study for the longitudinal component.

Judgment rules. Y = Yes; N = No; U = Unclear; NA = Not applicable. For analytical cross-sectional studies, item X2 was designated NA because eligibility in these general-population samples was not based on diagnosis of a health condition. Where a report contributed more than one eligible neurocognitive outcome, outcome-measure judgments reflect the outcomes included in this review; when methods differed across eligible outcomes, the more conservative judgment was retained. No composite numerical quality score was calculated. Item-level judgments were used to identify methodological limitations relevant to synthesis and interpretation.

File S2A. JBI appraisal: cohort studies (n = 17)

Study	C1	C2	C3	C4	C5	C6	C7	C8	C9	C10	C11	Key methodological concern(s)
Linebarger & Walker [28]	Y	Y	Y	Y	Y	Y	Y	Y	U	U	Y	Caregiver viewing logs; attrition and missing-data handling not fully reported.
Barr et al. [29]	Y	Y	Y	Y	Y	Y	Y	Y	U	U	Y	Small longitudinal cohort; follow-up and attrition handling incompletely reported.
Duch et al. [30]	Y	Y	U	Y	Y	Y	Y	Y	U	U	Y	Caregiver-reported 24-h recall; attrition and missing-data handling unclear.
Madigan et al. [31]	Y	Y	U	Y	Y	Y	Y	Y	Y	Y	Y	Caregiver-reported screen time; otherwise strong repeated longitudinal modelling.
Supanitayanon et al. [33]	Y	Y	U	Y	Y	Y	Y	Y	U	U	Y	Caregiver-reported screen exposure; attrition and missing-data handling unclear.
Martinot et al. [35]	Y	Y	U	Y	Y	Y	Y	Y	U	U	Y	Caregiver-reported exposure; residual confounding and attrition remain possible.
Dore et al. [41]	Y	Y	Y	Y	Y	U	Y	Y	U	U	Y	Time diary was collected near the end of the skill-gain interval, limiting temporal precedence; attrition handling unclear.
Yamamoto et al. [43]	Y	Y	U	Y	Y	Y	Y	Y	Y	Y	Y	Caregiver-reported screen time; large cohort and adjusted longitudinal analyses mitigate other concerns.
Kracht et al. [44]	Y	Y	U	Y	Y	Y	Y	Y	U	U	Y	Small longitudinal cohort; caregiver-reported exposure; incomplete-follow-up handling unclear.
Takahashi et al. [45]	Y	Y	U	Y	Y	Y	Y	Y	Y	Y	Y	Caregiver-reported exposure; otherwise large prospective cohort with adjusted analyses.
Binet et al. [47]	Y	Y	U	Y	Y	Y	Y	Y	Y	U	Y	Caregiver-reported exposure; approximately 79% retention; incomplete-follow-up adjustment not clearly reported.
Yang et al. [49]	Y	Y	U	Y	Y	Y	Y	Y	Y	Y	Y	Caregiver-reported exposure; repeated prospective measures and explicit missing-data strategy.
Gath et al. [51]	Y	Y	U	Y	Y	Y	Y	Y	U	U	Y	Caregiver-reported exposure; attrition and missing-data handling not fully clear.
McHarg et al. [62]	Y	Y	U	Y	Y	Y	Y	Y	Y	Y	Y	Caregiver-reported screen exposure; direct executive-function assessment and explicit missing-data strategy.
Corkin et al. [63]	Y	Y	U	Y	Y	Y	Y	Y	U	U	Y	Caregiver-reported exposure; attrition and missing-data handling incompletely reported.
Portugal et al. [68]	Y	Y	U	Y	Y	Y	Y	Y	Y	U	Y	Parent-reported touchscreen exposure and small sample; attrition described, but incomplete-follow-up adjustment unclear.
Fitzpatrick et al. [70]	Y	Y	U	Y	Y	Y	Y	Y	U	U	Y	Parent-reported screen trajectories; attrition and missing-data handling not fully clear.

File S2B. JBI appraisal: analytical cross-sectional studies (n = 22)

Study	X1	X2	X3	X4	X5	X6	X7	X8	Key methodological concern(s)
Hutton et al. [32]	Y	NA	Y	Y	Y	Y	Y	Y	Cross-sectional design limits temporal inference; small neuroimaging sample.
Sundqvist et al. [34]	Y	NA	U	Y	Y	Y	Y	Y	Caregiver-reported digital-media exposure; cross-sectional temporal ambiguity.
John et al. [36]	Y	NA	U	U	Y	Y	Y	Y	Caregiver-reported exposure and parent-reported developmental outcome; cross-sectional design.
Axelsson et al. [37]	Y	NA	U	Y	Y	Y	Y	Y	Caregiver-reported media exposure; observational temporal ambiguity.
McArthur et al. [38]	Y	NA	U	Y	Y	Y	Y	Y	Main exposure and outcomes were concurrent at 36 months despite adjustment for earlier developmental performance.
Mustonen et al. [39]	Y	NA	U	Y	Y	Y	Y	Y	Caregiver-reported exposure; cross-sectional temporal ambiguity.
Medawar et al. [40]	Y	NA	U	Y	Y	Y	Y	Y	Online/caregiver-reported exposure; cross-sectional design.
Rai et al. [42]	Y	NA	Y	Y	Y	Y	Y	Y	Small pilot sample, although repeated diary exposure and direct assessments reduce measurement concerns.
Monteiro et al. [46]	Y	NA	Y	Y	Y	Y	Y	Y	Cross-sectional exploratory sample; validated ScreenQ and language assessment support measurement validity.

Study	X1	X2	X3	X4	X5	X6	X7	X8	Key methodological concern(s)
Rayce et al. [48]	Y	NA	U	Y	Y	Y	Y	Y	Parent-reported typical-day mobile screen time; cross-sectional design.
Gago-Galvagno et al. [50]	Y	NA	U	Y	U	U	Y	Y	Very small non-probability sample; confounding control not clearly established; caregiver-reported exposure.
Linebarger et al. [53]	Y	NA	U	Y	Y	Y	Y	Y	Caregiver-reported media use; cross-sectional associations despite multivariable modelling.
Nathanson et al. [54]	Y	NA	U	Y	Y	Y	Y	Y	Parent-reported television exposure; cross-sectional temporal ambiguity.
Munzer et al. [57]	Y	NA	U	Y	Y	Y	Y	Y	Parent-reported media exposure; cross-sectional design.
Tamana et al. [58]	Y	NA	U	Y	Y	Y	Y	Y	Primary age-5 association was concurrent and screen exposure was parent-reported, although rich confounder adjustment was used.
Yang et al. [60]	Y	NA	U	Y	Y	Y	Y	Y	Parent-reported electronic-game exposure; cross-sectional design.
Jusienė et al. [61]	Y	NA	U	Y	Y	Y	Y	Y	Parent-reported screen exposure; cross-sectional design.
Konok et al. [64]	Y	NA	U	Y	U	U	Y	Y	Observational component used in this review is cross-sectional; exposure validity and confounder control are not fully clear.
McNeill et al. [65]	Y	NA	U	Y	Y	Y	Y	Y	Parent-reported application/program use; cross-sectional design.
Nichols [66]	Y	NA	Y	Y	Y	Y	Y	Y	Cross-sectional temporal ambiguity despite detailed 24-h diary exposure and weighted analyses.
Choe et al. [67]	Y	NA	U	Y	Y	Y	Y	Y	Small multi-method cross-sectional sample; parent-reported screen exposure.
Horowitz-Kraus et al. [69]	Y	NA	U	Y	Y	Y	Y	Y	Cross-sectional design and parent-reported screen time; direct EEG/attention outcomes reduce outcome-measure concerns.

File S2C. JBI appraisal: randomized controlled trials (n = 4)

Study	R1	R2	R3	R4	R5	R6	R7	R8	R9	R10	R11	R12	R13	Key methodological concern(s)
Lillard & Peterson [52]	Y	N	Y	N	N	Y	N	Y	Y	Y	Y	Y	Y	Allocation concealment absent; participant/provider blinding infeasible; outcome-assessor blinding incomplete.
Lillard et al. [55]	Y	U	U	N	N	Y	U	Y	Y	Y	Y	Y	Y	Allocation concealment, baseline equivalence and assessor blinding incompletely reported; participant/provider blinding infeasible.
Huber et al. [56]	Y	U	U	N	N	Y	U	Y	Y	Y	N	Y	Y	Allocation concealment/baseline balance/assessor blinding unclear; post-randomization exclusions without clear intention-to-treat analysis.
Li et al. [59]	Y	U	Y	N	N	Y	U	Y	Y	Y	U	Y	Y	Allocation concealment and assessor blinding unclear; participant/provider blinding infeasible; handling of exclusions across experiments not fully clear.

JBI item definitions used in Files S2A–S2C

Cohort studies	Analytical cross-sectional studies	Randomized controlled trials
C1. Were the two groups similar and recruited from the same population?	X1. Were the criteria for inclusion in the sample clearly defined?	R1. Was true randomization used for assignment of participants to treatment groups?
C2. Were the exposures measured similarly to assign people to both exposed and unexposed groups?	X2. Were objective, standard criteria used for measurement of the condition?	R2. Was allocation to treatment groups concealed?
C3. Was the exposure measured in a valid and reliable way?	X3. Was the exposure measured in a valid and reliable way?	R3. Were treatment groups similar at baseline?
C4. Were confounding factors identified?	X4. Were the outcomes measured in a valid and reliable way?	R4. Were participants blind to treatment assignment?
C5. Were strategies to deal with confounding factors stated?	X5. Were confounding factors identified?	R5. Were those delivering treatment blind to treatment assignment?
C6. Were the groups/participants free of the outcome at the start of the study (or at the moment of exposure)?	X6. Were strategies to deal with confounding factors stated?	R6. Were treatment groups treated identically other than the intervention of interest?
C7. Were the outcomes measured in a valid and reliable way?	X7. Was appropriate statistical analysis used?	R7. Were outcome assessors blind to treatment assignment?
C8. Was the follow-up time reported and sufficient to be long enough for outcomes to occur?	X8. Were the study subjects and the setting described in detail?	R8. Were outcomes measured in the same way for treatment groups?
C9. Was follow-up complete, and if not, were reasons for loss to follow-up described and explored?		R9. Were outcomes measured in a reliable way?
C10. Were strategies to address incomplete follow-up utilized?		R10. Was follow-up complete and, if not, were differences between groups in follow-up adequately described and analyzed?
C11. Was appropriate statistical analysis used?		R11. Were participants analyzed in the groups to which they were randomized?
		R12. Was appropriate statistical analysis used?
		R13. Was the trial design appropriate, and were any deviations from the standard RCT design accounted for in conduct and analysis?

Summary of methodological patterns identified by the JBI appraisal

Across the 17 cohort studies, the principal recurring concern was exposure measurement: 14/17 received an Unclear judgment for C3 because screen exposure was primarily derived from caregiver report without an objective device-based validation measure. Follow-up completeness and handling of missing follow-up data were also incompletely reported in several studies (C9 and C10). In contrast, confounding was generally identified and addressed, neurocognitive outcomes were usually measured with standardized or otherwise appropriate instruments, and statistical analyses were judged appropriate.

Across the 22 analytical cross-sectional studies, 18/22 received an Unclear judgment for X3 because screen exposure was usually parent- or caregiver-reported. The cross-sectional nature of these analyses also limits temporal inference even where confounder adjustment was adequate. Neurocognitive outcome measurement and statistical analysis were generally appropriate. Two small studies had insufficiently clear control of confounding, and one study relied primarily on a parent-reported developmental outcome, resulting in more cautious judgments.

Across the four randomized experimental studies, true randomization was reported, but allocation concealment was absent or insufficiently described. Blinding of young participants and personnel delivering the media exposure was generally infeasible because the experimental conditions were perceptibly different. Outcome-assessor blinding was absent or incompletely reported in several experiments. Outcome measurement and statistical methods were otherwise generally appropriate, although one study excluded randomized participants without a clearly reported intention-to-treat analysis.

Overall interpretation. These findings support greater confidence in the repeated direction of associations when they arise in larger prospective cohorts with standardized neurocognitive outcomes and extensive covariate adjustment, but they also support cautious causal interpretation. The evidence base remains vulnerable to exposure misclassification from caregiver-reported screen use, residual confounding, reverse causality in cross-sectional analyses, and incomplete reporting of attrition or allocation procedures in some studies.

JBI appraisal tools

Barker TH, Hasanoff S, Aromataris E, Stone JC, Leonardi-Bee J, Sears K, et al. The revised JBI critical appraisal tool for the assessment of risk of bias for analytical cross-sectional studies. JBI Evidence Synthesis. 2026;24(3):401–408.

Barker TH, Hasanoff S, Aromataris E, Stone JC, Leonardi-Bee J, Sears K, et al. The revised JBI critical appraisal tool for the assessment of risk of bias for cohort studies. JBI Evidence Synthesis. 2025;23(3):441–453. Supplemental Digital Content 1. doi:10.11124/JBIES-24-00103.

Barker TH, Stone JC, Sears K, Klugar M, Tufanaru C, Leonardi-Bee J, Aromataris E, Munn Z. The revised JBI critical appraisal tool for the assessment of risk of bias for randomized controlled trials. JBI Evidence Synthesis. 2023;21(3):494–506.
