# Supplementary material for: Associations Between Early Screen Exposure and Neurocognitive Outcomes in Children Aged 2–5 Years: A Systematic Review
Source: Healthcare (Basel). 2026 Sep 19;14(18):3081. doi: 10.3390/healthcare14183081 (PMC13606092; doi:10.3390/healthcare14183081)
Supplement: Supplementary file 1 [file healthcare-14-03081-s001.zip › Supplementary_Table_S2_Database_Search_Strategies.pdf]

Supplementary Table S2. Complete Database-Specific Search Strategies

Searches were conducted in PubMed, Web of Science, and Scopus. The search concepts were combined with AND, while synonymous terms within each concept were combined with OR. The final database searches were conducted on 24 June 2026. Eligibility was restricted to English-language peer-reviewed quantitative studies published from 1 January 2000 to 31 December 2025; peer-review status was verified during screening rather than used as a database search filter.

Database	Search field / syntax	Complete search strategy	Limits / filters
PubMed	Title/Abstract [tiab]	(<br>("screen time"[Title/Abstract] OR "screen exposure"[Title/Abstract] OR "digital media"[Title/Abstract] OR television[Title/Abstract] OR TV[Title/Abstract] OR smartphone[Title/Abstract] OR tablet[Title/Abstract] OR computer[Title/Abstract] OR "background television"[Title/Abstract] OR "background media"[Title/Abstract])<br>AND<br>("early childhood"[Title/Abstract] OR "preschool child"[Title/Abstract] OR preschooler[Title/Abstract] OR toddler[Title/Abstract] OR "young child"[Title/Abstract] OR "children aged 2-5 years"[Title/Abstract])<br>AND<br>("language development"[Title/Abstract] OR vocabulary[Title/Abstract] OR "cognitive development"[Title/Abstract] OR cognition[Title/Abstract] OR "executive function"[Title/Abstract] OR attention[Title/Abstract] OR "inhibitory control"[Title/Abstract] OR "working memory"[Title/Abstract] OR "self-regulation"[Title/Abstract] OR "developmental outcome"[Title/Abstract])<br>)	Language: English.<br>Publication date: 2000/01/01–2025/12/31.<br>Search date: 24 June 2026.
Web of Science	Topic search (TS=)	TS=(<br>("screen time" OR "screen exposure" OR "digital media" OR television OR TV OR smartphone OR tablet OR computer OR "background television" OR "background media")<br>AND<br>("early childhood" OR "preschool child" OR preschooler OR toddler OR "young child" OR "children aged 2-5 years")<br>AND<br>("language development" OR vocabulary OR "cognitive development" OR cognition OR "executive function" OR attention OR "inhibitory control" OR "working memory" OR "self-regulation" OR "developmental outcome")<br>)	Language filter: English.<br>Publication years: 2000–2025.<br>Search date: 24 June 2026.
Scopus	TITLE-ABS-KEY()	TITLE-ABS-KEY(<br>("screen time" OR "screen exposure" OR "digital media" OR television OR TV OR smartphone OR tablet OR computer OR "background television" OR "background media")<br>AND<br>("early childhood" OR "preschool child" OR preschooler OR toddler OR "young child" OR "children aged 2-5 years")<br>AND<br>("language development" OR vocabulary OR "cognitive development" OR cognition OR "executive function" OR attention OR "inhibitory control" OR "working memory" OR "self-regulation" OR "developmental outcome")<br>)	Language filter: English.<br>Publication years: 2000–2025.<br>Search date: 24 June 2026.

Notes. PubMed searches title, abstract, and author-keyword content. Web of Science Topic searches title, abstract, author keywords, and Keywords Plus. Scopus searches article title, abstract, and keywords. The search strings above operationalize the search concepts reported in Section 2.3 of the manuscript.
