# Supplementary material for: Associations Between Early Screen Exposure and Neurocognitive Outcomes in Children Aged 2–5 Years: A Systematic Review
Source: Healthcare (Basel). 2026 Sep 19;14(18):3081. doi: 10.3390/healthcare14183081 (PMC13606092; doi:10.3390/healthcare14183081)
Supplement: Supplementary file 1 [file healthcare-14-03081-s001.zip › Supplementary_Table_S3_Study-Level_Quantitative_Findings.pdf]

Supplementary Table S3. Study-Level Quantitative Findings of the Included Studies

Table note. Reference numbers correspond to the main manuscript. The table presents representative quantitative findings most directly relevant to the review question; it is not intended to reproduce every model reported in each primary study. Effect estimates are retained in the metric used by the original publication and were not standardized or pooled because of substantial heterogeneity in study design, exposure definitions, comparison groups, outcome measures, and statistical approaches. Where a study reported multiple programme-, task-, or context-specific analyses without a single meaningful summary estimate, the principal inferential pattern is reported. Country/study setting identifies the geographic setting reported by the primary study and should not be interpreted as participant nationality. Specific ethnic or population descriptors are included only when explicitly reported. The review's English-language criterion concerned publication language only.

Study	Country / study setting	Sample size	Exposure / comparator	Neurocognitive outcome(s)	Statistical measure(s)	Principal quantitative finding(s)
**Linebarger and Walker, 2005 [28]**	**United States; greater Kansas City metropolitan area and Lawrence, Kansas; community/childcare-center sample**	51	Repeated parent viewing logs from 6 months; programme/content-specific television exposure	Vocabulary; expressive language	Hierarchical linear models; programme-specific associations	At 30 months, associations differed by programme. Dora the Explorer, Blue's Clues, Arthur, Clifford, and Dragon Tales were associated with larger vocabularies and higher expressive-language scores; Teletubbies with fewer vocabulary words and smaller expressive-language scores; Sesame Street with smaller expressive-language scores; Barney & Friends with fewer vocabulary words but more expressive language. No single pooled programme coefficient was reported.
**Barr et al., 2010 [29]**	**United States; Georgetown University longitudinal community sample**	60	24-h television diaries at ages 1 and 4; adult-directed vs child-directed programming; household TV	Cognitive skills; school readiness; EF	Regression analyses; content-specific associations	High adult-directed television exposure in infancy and at age 4, and high household television use at age 4, were associated with poorer EF. High adult-directed exposure during the preschool period was also associated with poorer cognitive outcomes; child-directed exposure was not associated with the age-4 outcomes. No single summary coefficient was reported.
**Duch et al., 2013 [30]**	**United States; urban Early Head Start program; Hispanic infants/toddlers**	119	>2 h/day vs <=2 h/day; 24-h recall; child-directed vs adult-directed media	ASQ-3 communication/language	Logistic regression	Mean child screen exposure was 3.29 h/day (median 2.5). >2 h/day was associated with approximately 5.5-fold higher odds of low communication scores; adjusted logit coefficient beta=-1.65, p=.04. Child-directed exposure >2 h/day: adjusted beta=-1.81, p=.009. Longitudinally, high total television exposure remained associated with lower communication scores (beta=-1.49, p=.008).
**Madigan et al., 2019 [31]**	**Canada; Calgary, Alberta; All Our Families cohort**	2,441	Parent-reported total screen time at 24 and 36 months	ASQ-3 developmental screening	Cross-lagged standardized beta; 95% CI	Higher screen time at 24 months predicted lower developmental scores at 36 months (beta=-0.06, 95% CI -0.10 to -0.01), and higher screen time at 36 months predicted lower scores at 60 months (beta=-0.08, 95% CI -0.13 to -0.02).
**Hutton et al., 2020 [32]**	**United States; Cincinnati, Ohio; children's hospital and community primary-care clinics**	47 (DTI)	ScreenQ composite of screen-based media use	Emergent literacy/language; white-matter integrity	Linear models; R2; F; p	Higher ScreenQ scores were associated with lower EVT-2 scores (F(2,43)=5.14, R2=.19, p<.01), lower CTOPP-2 scores (F(2,35)=6.64, R2=.28, p<.01), and lower GRTR scores (F(2,44)=17.08, R2=.44, p<.01). Higher ScreenQ was also associated with lower FA and higher RD in language/literacy tracts (family-wise error corrected p<.05).
**Supanitayanon et al., 2020 [33]**	**Thailand; Bangkok; Chulalongkorn University longitudinal cohort**	274	Screen-media onset and cumulative high-exposure months in first 2 years	Preschool cognitive development	Multiple regression; beta; p	At age 2, cognitive performance was associated with later screen onset (beta=.113, p<.05), fewer months of high screen exposure (beta=.282, p<.001), and more months of verbal caregiver interaction (beta=.261, p<.001). Earlier onset and greater cumulative high exposure were therefore linked to less favourable cognitive performance.
**Sundqvist et al., 2021 [34]**	**Sweden; Linkoping municipal area; community sample of Swedish-speaking families**	92 (analysis N varied)	Digital-media exposure/content and parental device use during routines	Vocabulary; grammar; pragmatics	Multiple regression; adjusted R2	Vocabulary model: F(3,69)=9.87, p<.001, adjusted R2=.27; turn-taking accounted for 16% of variance, TV-content variables 9%, and parental device use during routines 5%. Grammar model: F(2,70)=6.68, p<.01, adjusted R2=.14. Pragmatics model: F(3,69)=6.92, p<.001, adjusted R2=.20.
**Martinot et al., 2021 [35]**	**France; EDEN Mother-Child Cohort**	1,562	Total screen time and television exposure during family meals	Language development; verbal IQ	Adjusted mean differences; 95% CI	Television always on during family meals at age 2 vs never was associated with a 5.9-point lower CDI score (95% CI -11.7 to -0.1) and a 3.2-point lower verbal-IQ score at 5-6 years (95% CI -6.0 to -0.3; trend p=.002). Associations with overall daily screen duration were substantially weaker.
**John et al., 2021 [36]**	**India; Thiruvalla, Kerala; two kindergarten schools**	189	>1 h/day vs <=1 h/day; supervision/context of use	Parent-reported attention, intelligence, social interaction	Odds ratios; 95% CI	Excess screen time itself was not significantly associated with suspected deficits: attention OR=1.2 (95% CI 0.3-5.6), intelligence OR=0.8 (0.2-3.6), social interaction OR=0.5 (0.1-2.6). Inconsistent supervision was associated with attention OR=3.2 (1.3-8.2), intelligence OR=4.1 (1.3-13.3), and social-interaction OR=15.3 (1.9-121.2).
**Axelsson et al., 2022 [37]**	**Australia; New South Wales; University of Newcastle-affiliated preschool sample**	101 (outcome N=91-97)	Duration of entertainment screen content; with someone vs alone	Communication; problem solving; attention	Regression; standardized estimates; p	Entertainment content averaged about 2 h/day. Longer entertainment exposure predicted poorer problem solving (standardized estimate=.35, p=.004) and more attention problems (standardized estimate=.31, p=.010). The duration-by-viewing-context interaction for problem solving was also significant (standardized estimate=-.65, p=.007). Communication scores were likewise lower with greater exposure.
**McArthur et al., 2022 [38]**	**Canada; Calgary, Alberta; All Our Families cohort**	1,994	<=1 h/day vs 2 h/day vs >=3 h/day	Developmental milestones; vocabulary; behaviour	Adjusted odds ratios; 95% CI	Compared with <=1 h/day (n=935), 2 h/day (n=725) and >=3 h/day (n=333) were associated with behavioural problems (AOR 1.30-1.90) and delayed developmental milestones (AOR 1.41-1.68). For vocabulary, >=3 h/day vs <=1 h/day: AOR=1.94 (95% CI 1.29-2.91); 2 h/day was not significant (AOR=1.16, 0.84-1.62).

Study	Country / study setting	Sample size	Exposure / comparator	Neurocognitive outcome(s)	Statistical measure(s)	Principal quantitative finding(s)
**Mustonen et al., 2022 [39]**	**Finland; Helsinki capital area daycare centers; monolingual Finnish-speaking children**	164	Child screen time alone, co-viewing, and maternal screen time	Vocabulary; total language	Multiple regression; B; p	Child screen time alone predicted lower vocabulary (B=-0.08, p=.03) and lower total-language scores (B=-0.13, p=.02). Maternal screen time also predicted lower vocabulary (B=-0.02, p=.01) and total-language scores (B=-0.04, p=.007).
**Medawar et al., 2023 [40]**	**Argentina; community/online sample of Argentinean toddlers**	465	TV/screen exposure; educational content; joint engagement/scaffolding	Vocabulary; sentence use	Multivariable regression	TV exposure contributed negatively to vocabulary, and TV exposure together with educational-content exposure contributed negatively to sentence use. Home-literacy beliefs, parent-child time, and verbal scaffolding contributed positively. The study reported multiple model-specific coefficients rather than one summary effect estimate.
**Dore et al., 2023 [41]**	**United States; Ohio; preschool time-diary study**	179	24-h time diary: total, nighttime, educational, and joint media use	Academic/school-readiness skills; social-behavioural skill gains	Longitudinal skill-gain models	Very high total media use was associated with smaller gains in selected social skills, and high nighttime media use with smaller assertiveness gains. Quantity of media use was not associated with gains in language, literacy, or mathematics, highlighting non-uniform associations across outcomes.
**Rai et al., 2023 [42]**	**Canada; Edmonton, Alberta and surrounding areas; pilot virtual study**	44	2-week diary: total/video/educational screen time and co-use	Working memory; inhibitory/self-control; vocabulary	Spearman rho; p	Total screen time correlated with poorer working memory (rho=-.40, p=.011), as did video viewing (rho=-.42, p=.007). After adjustment, these associations were attenuated (rho=-.32, p=.056). Educational screen time correlated with vocabulary (rho=.38, p=.018), whereas co-use correlated with lower self-control (rho=-.32, p=.049).
**Yamamoto et al., 2023 [43]**	**Japan; Japan Environment and Children's Study (JECS)**	57,980	Screen time at ages 1 and 2 years	Developmental performance	Cross-lagged standardized beta; 95% CI	Screen time at age 1 predicted lower developmental performance at age 2 (beta=-.05, 95% CI -.06 to -.04), and screen time at age 2 predicted lower development at age 3 (beta=-.08, 95% CI -.09 to -.06). Reverse paths from communication to later screen time were also observed (approximately beta=-.03 to -.06), supporting bidirectionality.
**Kracht et al., 2023 [44]**	**United States; Arkansas; GLOWING prospective cohort**	89	Child screen time at 3, 12, and 24 months; WHO guideline categories	Bayley-III cognitive/language development	Correlations; adjusted regression	In unadjusted analyses, girls' screen time at 3 months correlated with lower cognitive scores at 24 months (r=-.29, p=.06); boys' screen time at 3 months correlated with lower language scores (r=-.25, p=.08). Few developmental associations persisted after adjustment; authors reported no other significant screen-development associations (p>.05).
**Takahashi et al., 2023 [45]**	**Japan; Tohoku Medical Megabank Birth and Three-Generation Cohort, Miyagi/Sendai**	7,097	Screen time at age 1: <1, 1-<2, 2-<4, >=4 h/day	Communication and problem-solving delay at ages 2 and 4	Adjusted OR; 95% CI	At age 2, >=4 vs <1 h/day was associated with communication delay OR=4.78 (95% CI 3.24-7.06) and problem-solving delay OR=2.67 (1.72-4.14). At age 4, corresponding ORs were 2.68 (1.68-4.27) for communication and 1.91 (1.17-3.14) for problem solving.
**Monteiro et al., 2024 [46]**	**Portugal; Portuguese preschool sample**	237	ScreenQ digital-media-use score	Expressive language (semantic and morphosyntactic)	Correlation and multiple regression	ScreenQ was significantly negatively correlated with verbal oral expression and remained significant in the multivariable regression model; parental education was the strongest predictor. The publication reports domain/model-specific coefficients rather than a single summary estimate in the abstract.
**Binet et al., 2024 [47]**	**Canada; Nova Scotia community preschool cohort during the COVID-19 pandemic**	315 (analysis N varied)	Pandemic-era preschool screen time; intensive >4 h/day vs light <1 h/day	Developmental milestones/global development	OR; beta; p	Intensive (>4 h/day) vs light (<1 h/day) screen exposure was associated with higher odds of global developmental delay (OR=4.29, p=.020). Mobile-device use was associated with a lower global developmental score (beta=-3.064, p=.028).
**Rayce et al., 2024 [48]**	**Denmark; national TRACES survey of 2- and 3-year-old children**	31,125 (fully adjusted N=28,141)	Mobile-device use: none, <1, 1-2, >2 h/day	Receptive/comprehension and expressive language	Adjusted OR; 95% CI	Compared with no mobile-device use, 1-2 h/day was associated with poorer comprehension (AOR=1.30, 95% CI 1.13-1.49) and expressive language (AOR=1.19, 1.03-1.37). >2 h/day: comprehension AOR=1.42 (1.08-1.88), expressive language AOR=1.46 (1.10-1.93).
**Yang et al., 2024 [49]**	**France; ELFE national birth cohort**	13,763	Daily screen time and television during family meals	Cognitive/language development	Adjusted mean differences per exposure; 95% CI	TV always on during family meals at age 2 was associated with lower language-related scores (MacArthur-Bates: -1.67, 95% CI -2.21 to -1.13; later CDI: -0.82, -1.13 to -0.33). At 3.5 years, each additional screen hour/day was associated with CDI -0.67 points (95% CI -0.94 to -0.40); the longitudinal association with CDI at 5.5 years was not significant (-0.31, -0.74 to 0.13).
**Gago-Galvagno et al., 2024 [50]**	**Argentina; Buenos Aires; toddler-parent dyads**	33 dyads	Screen use/background TV and dyadic interaction	Receptive/expressive communication; flexibility/regulation	Pearson r; p	Background television correlated with poorer receptive communication (r=-.463, p<.01), expressive communication (r=-.533, p<.01), and flexibility (r=-.492, p<.01). Associations for television/device use were otherwise less consistent across core outcomes.
**Gath et al., 2025 [51]**	**New Zealand; Growing Up in New Zealand cohort**	6,281	Direct screen time at age 2; later preschool outcomes	Language; early educational skills; peer functioning	Adjusted regression/estimated means	Direct screen time >1.5 h/day at age 2 was associated with below-average language/educational outcomes at 4.5 years. Adjusted language means showed a graded pattern: 0 h/day=0.15; 1 h=.09; 2 h=-.06; 3 h=-.19; 4 h=-.30; 5 h=-.36; >=6 h=-.41 (standardized outcome scale).
**Lillard and Peterson, 2011 [52]**	**United States; Charlottesville, Virginia; laboratory preschool sample**	60 (20/group)	9 min fast-paced fantastical cartoon vs educational cartoon vs drawing	Composite EF; delay of gratification	ANCOVA/regression; p; partial eta2	Intervention effect on composite EF: p=.01, partial eta2=.15; fast-paced group performed worse than drawing (p=.004) and differed marginally from educational TV (p=.05). Delay-of-gratification effect: p=.03, partial eta2=.12; fast-paced group waited less than drawing (p=.03) and educational TV (p=.02).

Study	Country / study setting	Sample size	Exposure / comparator	Neurocognitive outcome(s)	Statistical measure(s)	Principal quantitative finding(s)
**Linebarger et al., 2014 [53]**	**United States; nationally representative caregiver telephone survey**	1,156 overall (preschool subgroup reported separately)	24-h media diary; foreground/background TV; content; cumulative risk	Parent-reported executive functioning	Regression/moderation	Effects varied by developmental stage, content, context, and cumulative risk. In preschoolers, media-context/cumulative-risk interactions were reported rather than a uniform exposure effect; programme content contributed additional explained variance in EF in related models. No single overall screen-time coefficient adequately represents the study.
**Nathanson et al., 2014 [54]**	**United States; Ohio; preschool community sample**	107	Cumulative TV viewing/background TV; content genres; age of onset	Composite executive function	Hierarchical regression; standardized beta; R2	Adding TV-viewing variables increased explained variance. Cumulative TV viewing predicted poorer EF (standardized beta approximately -.36 to -.37, p<.01 across models), while PBS viewing predicted better EF (beta=.23, p<.01). Models explained approximately 44%-47% of EF variance.
**Lillard et al., 2015 [55]**	**United States; University of Virginia laboratory experiments**	300 across 3 studies (160/60/80)	10-20 min fast/fantastical vs slow/realistic TV, book, or play	Immediate executive function	Experimental group comparisons	Study 1 (N=160): two fast/fantastical shows produced lower EF than slow/realistic TV or play. Study 2 (N=60): fast/fantastical educational and entertainment TV produced lower EF than reading. Study 3 (N=80): fantasy, rather than pacing alone, accounted for the immediate EF disruption.
**Huber et al., 2018 [56]**	**Australia; Melbourne, Victoria; Swinburne University of Technology preschool sample**	96	Educational app vs educational TV vs cartoon content	Executive function; delay of gratification; working memory	Experimental group comparisons	Children using the educational app showed greater delay of gratification than the cartoon group, and working-memory performance improved in selected comparisons. Effects varied by media type, indicating no uniform adverse effect of screen exposure.
**Munzer et al., 2018 [57]**	**United States; Michigan; Growing Healthy study (pre-intervention sample)**	541	Daily media exposure; background TV; TV during meals	Delay of gratification/self-regulation; temperament	Adjusted beta; 95% CI	Each additional hour/day of media exposure was associated with 10.30 s less delay-of-gratification waiting (beta=-10.30, 95% CI -19.35 to -1.25). Each one-unit increase in background-TV exposure was associated with 12.63 s less waiting (beta=-12.63, 95% CI -24.34 to -0.93).
**Tamana et al., 2019 [58]**	**Canada; Canadian Healthy Infant Longitudinal Development (CHILD) birth cohort**	2,427 (screen N=2,322)	>2 h/day vs <30 min/day	Inattention/ADHD symptoms; externalizing behaviour	Adjusted OR and adjusted mean difference; 95% CI	>2 h/day was associated with a 2.2-point higher externalizing T-score (95% CI .9-3.5), clinically significant inattention OR=5.9 (95% CI 1.6-21.5), and meeting ADHD criteria OR=7.7 (1.6-38.1), compared with <30 min/day.
**Li et al., 2020 [59]**	**China; urban public preschool in central China**	130 across 3 experiments (90/20/20)	Fantastical animated content; short-term experimental exposure	Immediate executive function; PFC EEG	t test; Cohen d; p	In Experiment 2, high-fantasy content produced lower EF than the comparison condition (t(18)=-2.51, p<.05, d=.31). Early PFC response also differed (t(19)=2.05, p=.05, d=.94). Effects were immediate and content-specific.
**Yang et al., 2020 [60]**	**China; Chinese preschool sample**	119	Electronic-game play duration/content	Executive functioning	Correlation and hierarchical regression	Time spent playing electronic games was positively, not negatively, related to overall EF (r=.26, p<.01). In regression, game time remained a positive predictor (B=.81, SE=.24, standardized beta=.19, p<.001; unique R2=.03). Action-game content showed a weak negative relation with inhibition but not overall EF.
**Jusiene et al., 2020 [61]**	**Lithuania; Vilnius; low-social-risk preschool sample**	190	TV/computer/smartphone/tablet use	Shifting; working memory; inhibition	Multiple regression	After adjustment, time spent with television, computer, smartphone, or tablet did not significantly predict shifting, working memory, or inhibition (all relevant p values non-significant). Findings varied by the type/context of media use rather than showing a uniform dose effect.
**McHarg et al., 2020 [62]**	**United Kingdom; British toddlers; Cambridge longitudinal study**	179 at 24 mo; 163 at 36 mo	Parent-reported screen time at 24 and 36 months	Executive functioning	Regression; standardized beta; p	Screen time increased from 86.86 min/day (SD 64.99) at 24 months to 116.18 min/day (SD 52.53) at 36 months (t(148)=6.53, p<.001). Screen time at 24 months predicted poorer EF at 36 months (beta=-.20, p=.035); concurrent 36-month screen time was not significant (p=.069).
**Corkin et al., 2021 [63]**	**New Zealand; Growing Up in New Zealand cohort**	3,787	Weekday TV exposure; >2 h/day patterns; meals in front of TV; content/context	Hot/cool EF; inattention/hyperactivity	Binomial logistic regression	Greater total weekday TV exposure at age 2 predicted poorer hot EF at 4.5 years. Eating meals in front of TV at age 4 predicted poorer cool EF at 4.5 years. Associations depended on exposure context; no single summary OR was reported in the abstract.
**Konok et al., 2021 [64]**	**Hungary; Budapest; preschool sample**	40 (Study 1); 56 (Study 2)	Mobile-device use and experimental digital-game exposure	Divided/selective attention; theory of mind	Correlational and experimental group analyses	Greater mobile-device use was associated with local attentional precedence and poorer theory-of-mind performance. Experimental digital-game exposure shifted divided attention toward local precedence; fast-game exposure altered the typical selective/divided-attention pattern. Multiple task-specific statistics were reported rather than one summary effect.
**McNeill et al., 2021 [65]**	**Australia; Wollongong, New South Wales; preschool sample**	247	Application use and media-program viewing	Visual-spatial/phonological working memory; psychosocial outcomes	Regression B; mean difference; 95% CI; p	Total media use predicted lower visual-spatial working memory (B=-.001, 95% CI -.003 to -.000, p=.026), as did programme viewing (B=-.002, -.003 to -.000, p=.033). High-dose app users showed higher phonological working memory vs non-users (MD=.31, 95% CI .04-.58, p=.025).
**Nichols, 2022 [66]**	**United States; nationally representative caregiver telephone survey**	1,180 overall; preschool N=789	Background TV during specific activities	Executive functioning	Regression B; p; delta R2	In preschoolers, background TV while playing alone predicted poorer EF (B=.94, p<.05), whereas background TV during routines/chores predicted stronger EF (B=-1.05, p<.05). Cumulative risk predicted poorer EF (B=1.20, p<.01). Background-TV context explained additional variance (delta R2=.089).

Study	Country / study setting	Sample size	Exposure / comparator	Neurocognitive outcome(s)	Statistical measure(s)	Principal quantitative finding(s)
**Choe et al., 2022 [67]**	**United States; western United States; racially diverse preschool sample**	72	Child mobile-device use, TV use, parent media use, child dysregulation	Behavioural self-regulation	Path analysis	Child mobile-device use and TV use were both negatively associated with behavioural self-regulation, with the mobile-device association stronger than the TV association. Earlier dysregulation and parental screen use were mostly unrelated to children's media use/self-regulation; model-specific standardized paths were reported.
**Portugal et al., 2023 [68]**	**United Kingdom; London-based longitudinal touchscreen cohort**	46 (low use=19; high use=27)	High vs low touchscreen use	Working memory/cognitive flexibility; impulse/self-control	MANOVA/ANOVA; eta2; p	Overall multivariate group effect was not significant (F(2,43)=2.269, p=.116, eta2=.095). Working-memory/cognitive-flexibility composite differed (F(1,44)=4.498, p=.040, eta2=.093; low-use M=.57 vs high-use M=.43), whereas impulse/self-control did not (F(1,44)=.074, p=.787).
**Horowitz-Kraus et al., 2024 [69]**	**Israel; 4-year-old preschool EEG study**	NR in abstract	Parent-reported screen time	Behavioural EF and EEG (N200/P300) during attention/inhibition task	Correlations/ERP analyses	Longer screen time was associated with lower behavioural EF and with EEG indices of poorer attention/inhibitory control, including greater P300 amplitude/latency differences between incongruent and congruent conditions. The accessible abstract does not report the sample size or individual correlation coefficients.
**Fitzpatrick et al., 2025 [70]**	**Canada; Nova Scotia; community-based preschool cohort**	315	Screen-time trajectories: low (~0.9 h/day), medium (~3.0), high (~6.38)	Inhibitory control; cognitive flexibility; effortful control	Regression coefficients	Compared with the low trajectory, the medium trajectory was associated with lower inhibitory control (b=-5.24), cognitive flexibility (b=-4.50), and effortful control (b=-.41). The high trajectory showed larger differences for cognitive flexibility (b=-10.12) and effortful control (b=-.61); inhibitory control was also significantly lower.

Abbreviations: AOR, adjusted odds ratio; ASQ-3, Ages and Stages Questionnaire, Third Edition; CDI, Communicative Development Inventory; CI, confidence interval; DTI, diffusion tensor imaging; EF, executive function; EEG, electroencephalography; EVT-2, Expressive Vocabulary Test, Second Edition; FA, fractional anisotropy; GRTR, Get Ready to Read; HLM, hierarchical linear modelling; MD, mean difference; NR, not reported in the accessible abstract; OR, odds ratio; PFC, prefrontal cortex; RD, radial diffusivity; R2, coefficient of determination; ScreenQ, ScreenQ media-use measure.

Interpretive caution: Most estimates are observational associations and should not be interpreted as causal effects. Sample size may vary across analyses because of missing data or availability of specific outcome measures.
